# Viral microRNA regulation of Akt is necessary for reactivation of Human Cytomegalovirus from latency in CD34^+^ hematopoietic progenitor cells and humanized mice

**DOI:** 10.1371/journal.ppat.1012285

**Published:** 2024-12-11

**Authors:** Nicole L. Diggins, Andrew H. Pham, Jennifer Mitchell, Christopher J. Parkins, Luke Slind, Rebekah Turner, Byeong-Jae Lee, Andrew D. Yurochko, Patrizia Caposio, Jay A. Nelson, Meaghan H. Hancock

**Affiliations:** 1 Vaccine and Gene Therapy Institute, Oregon Health & Science University, Beaverton, Oregon, United States of America; 2 Department of Microbiology & Immunology, Center for Applied Immunology and Pathological Processes, Center for Emerging Viral Threats, Louisiana State University Health Sciences Center-Shreveport, Shreveport, Louisiana, United States of America; Leibniz Institute of Virology (LIV), GERMANY

## Abstract

Human cytomegalovirus (HCMV) actively manipulates cellular signaling pathways to benefit viral replication. Phosphatidyl-inositol 3-kinase (PI3K)/Akt signaling is an important negative regulator of HCMV replication, and during lytic infection the virus utilizes pUL38 to limit Akt phosphorylation and activity. During latency, PI3K/Akt signaling also limits virus replication, but how this is overcome at the time of reactivation is unknown. Virally encoded microRNAs (miRNAs) are a key component of the virus arsenal used to alter signaling during latency and reactivation. In the present study we show that three HCMV miRNAs (miR-UL36, miR-UL112 and miR-UL148D) downregulate Akt expression and attenuate downstream signaling, resulting in the activation of FOXO3a and enhanced internal promoter-driven IE transcription. A virus lacking expression of all three miRNAs is unable to reactivate from latency both in CD34^+^ hematopoietic progenitor cells and in a humanized mouse model of HCMV infection, however downregulating Akt restores the ability of the mutant virus to replicate. These findings highlight the negative role Akt signaling plays in HCMV replication in lytic and latent infection and how the virus has evolved miRNA-mediated countermeasures to promote successful reactivation.

## Author summary

Human cytomegalovirus (HCMV) infection results in lifelong persistence of the virus through the establishment of latency, and viral reactivation is a significant cause of morbidity and mortality in solid organ and stem cell transplant patients. HCMV latency is established in CD34^+^ hematopoietic progenitor cells (HPCs) where the virus manipulates cell signaling pathways to maintain the viral genome and remain poised to reinitiate gene expression under the appropriate conditions, although the molecular mechanisms surrounding these processes are poorly understood. HCMV encodes microRNAs (miRNAs) that modulate expression of hundreds of cellular and viral genes and play important roles in regulating signaling in HPCs. In this study, we show that HCMV miR-UL36, miR-UL112, and miR-UL148D coordinately inhibit Akt expression, activation, and downstream signaling through nonconventional mechanisms. A mutant lacking these miRNAs is unable to reactivate from latency, yet complementing Akt regulation restores the ability of the mutant virus to reactivate, pointing to an important role for miRNA-mediated inhibition of Akt to promote HCMV reactivation.

## Introduction

Human cytomegalovirus (HCMV) infects most of the world population and institutes lifelong persistence in the host through the establishment of latent infections [1]. Latency is defined by maintenance of the viral genome in the absence of new virus production and occurs in CD34^+^ hematopoietic progenitor cells (HPCs) in the bone marrow and CD14^+^ monocytes [2,3]. Latent infection is punctuated by sporadic reactivation events that are stringently controlled by robust T cell responses in immunocompetent hosts. However, HCMV reactivation remains a significant cause of morbidity and mortality in the immunocompromised, including solid organ and hematopoietic stem cell transplant recipients [4,5], and is the leading cause of viral congenital infection [6]. Given its clinical importance, understanding how the virus manipulates infected HPCs during latency and reactivation is essential for developing novel approaches to target the latent reservoir.

Due to their long co-evolution with their hosts, CMVs have become master regulators of their environment, significantly remodeling cellular processes to benefit the virus lifecycle. Latency occurs in cell types inherently sensitive to intra- and extracellular cues that can drive the cells to proliferate, differentiate or undergo apoptosis. Thus, the cellular environment must be carefully controlled by viral gene products for successful latent infection and timely reactivation. Virally encoded miRNAs have emerged as important regulators of cell signaling during latency and reactivation due to their non-immunogenic nature and ability to act as rheostats, controlling signaling from external and internal stimuli to aid the virus lifecycle [7–9]. Small changes to miRNA-modulated signaling pathways disrupt the careful balance necessary for latency and/or reactivation and can mediate significant phenotypic effects as evidenced by the importance of HCMV miRNA regulation of TGFβ, MAPK and RhoA signaling in CD34^+^ HPCs [10–13].

The phosphatidyl-inositol 3-kinase (PI3K)/Akt pathway is a central regulator of cell state in response to external stimuli and a common target for manipulation by viruses [14–21]. Recruitment and activation of PI3K downstream of receptor tyrosine kinases, cytokine receptors, or G protein-coupled receptors promotes the conversion of phosphoinositol 4,5-bisphosphate (PIP2) to phosphoinositol 3,4,5-triphosphate (PIP3), which in turn recruits the serine/threonine kinase Akt to the membrane where it is phosphorylated at T308 by PDK1 and subsequently at S473 by mTORC2. Fully active Akt then dissociates from the membrane and phosphorylates downstream substrates to mediate changes in cell homeostasis, including enhanced protein synthesis, differentiation and regulation of stress responses [22–26]. HCMV attachment and entry stimulates Akt phosphorylation [27–29], but this modification is rapidly diminished in permissive fibroblasts by pUL38 [30] and by US28 in monocytes [28]. The importance of diminished Akt activity in HCMV lytic infection was shown by Zhang et al [29], who demonstrated that expression of a constitutively active Akt impairs virus replication in human fibroblasts. However, Akt signaling is necessary to stimulate protein translation and so pUL38 also directly and indirectly activates mTORC2 to bypass the need for Akt-mediated phosphorylation [31–33], highlighting how HCMV re-wires signaling pathways to benefit virus replication during lytic infection of fibroblasts. Diminished Akt activity is also necessary to maintain FOXO3a nuclear localization during lytic infection of fibroblasts [29], which is normally inhibited by Akt-mediated phosphorylation [34]. FOXO3a is a transcription factor that regulates differentiation and stress responses and was recently shown to be a key Akt substrate necessary for efficient HCMV lytic replication in fibroblasts [29], although how FOXO3a aids in virus replication remains to be defined. In the context of latency, addition of PI3K or Akt inhibitors during latency enhances HCMV replication in bone marrow-derived CD34^+^ HPCs [35], suggesting that Akt signaling is also inhibitory to virus replication in primary hematopoietic cells. Intriguingly, FOXO3a binding sites in the HCMV major immediate early promoter are necessary for expression of immediate early genes and reactivation from latency in bone marrow-derived CD34^+^ HPCs [36], supporting the hypothesis that inactivation of Akt is a critical component of the reactivation process, although if and how this occurs has not been investigated.

Here we show that, indeed, Akt signaling is inhibitory to HCMV reactivation in CD34^+^ HPCs derived from human embryonic stem cells (hESCs). Furthermore, we demonstrate that three HCMV-encoded miRNAs (miR-UL36, miR-UL112-3p and miR-UL148D-3p) regulate Akt expression that ultimately contributes to altered downstream signaling, including FOXO3a activation and expression of FOXO3a-dependent viral transcripts. Moreover, we demonstrate that regulation of Akt is one function of the three HCMV miRNAs required for efficient reactivation from latency in hESC-derived CD34^+^ HPCs and for the first time demonstrate the importance of HCMV miRNAs in latency and reactivation *in vivo* using a humanized mouse model. These data highlight the intricate role played by Akt signaling during different aspects of the HCMV replication cycle and how viral miRNAs play an essential role in tipping the balance from latency to reactivation by modulating the outcome of Akt signaling.

## Results

### Akt signaling attenuates HCMV reactivation from latency in hESC-derived CD34^+^ HPCs

**Fig 1:**
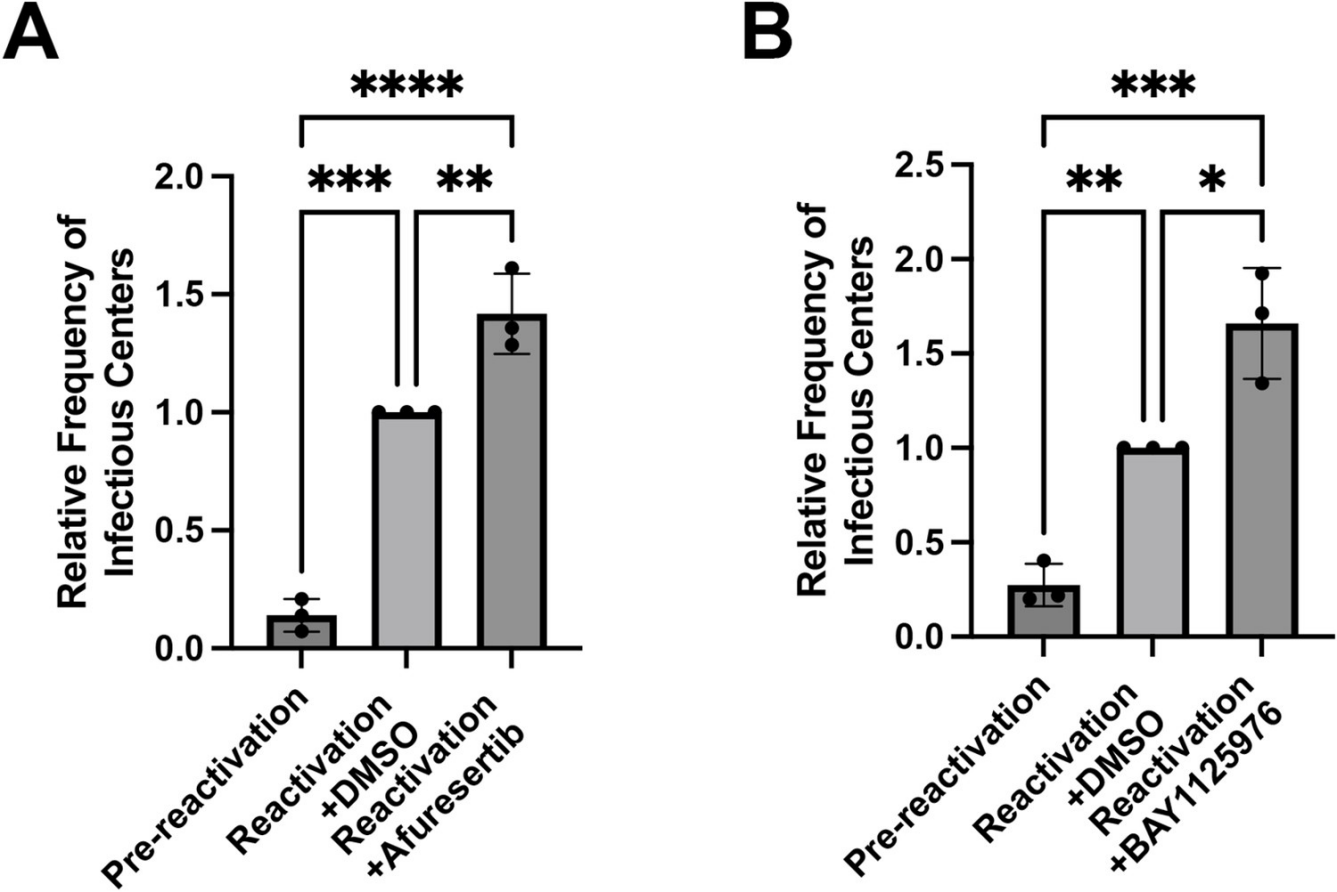
Akt restricts HCMV reactivation in hESC-derived CD34^+^ HPCs. hESC-derived CD34^+^ HPCs were infected with HCMV TB40/E-GFP at an MOI of 2 for 48hr and then sorted by FACS for viable, CD34^+^, GFP^+^ cells. Infected HPCs were maintained in LTBMC culture medium in transwells over stromal cells for 12 days to establish latency. Latently-infected cells were co-cultured with NHDFs in cytokine-rich media in the presence of Afuresertib (100 nM) (A), BAY1125976 (50nM) (B), or DMSO (control) in an extreme limiting dilution assay (ELDA) to measure virus reactivation [92]. An equal number of cells were mechanically disrupted and seeded in parallel to measure infectious virus present in the latency culture (pre-reactivation). Small molecule inhibitors were replenished at 7 days post-plating. At 21 days post-plating, the number of GFP^+^ wells were counted and the frequency of infectious center production was determined by ELDA software [93]. Reactivation is shown as the relative frequency of infectious centers compared to DMSO control-treated cells. (*p<0.02, **p<0.008, ***p<0.0005, ****p<0.0001 [one-way ANOVA with Tukey’s multiple comparison test]).

HCMV-mediated downregulation of Akt signaling is critical for efficient lytic replication, while intact Akt signaling prevents virus replication in bone marrow-derived CD34^+^ HPCs [29,35,36] and hESC-derived CD34^+^ HPCs (S1A Fig), suggesting that Akt acts to limit virus replication in multiple cell types during lytic and latent infection. At the time of reactivation, viral replication is re-initiated, but the role played by Akt and its effectors at this stage of the virus lifecycle is unknown. To address this question, we assessed the effects of Afuresertib, which inhibits the kinase activity of Akt [37,38], and BAY1125976, which inhibits Akt phosphorylation [39] on virus reactivation in hESC-derived CD34^+^ HPCs. In our hands, Afuresertib modestly inhibited Akt phosphorylation but clearly disrupted downstream Akt signaling in response to EGF treatment in fibroblasts (S1B–S1D Fig) while BAY1125976 inhibited phosphorylation of Akt at T308 and S473 in both HCMV-infected and uninfected fibroblasts (S2A and S2B Fig). To ensure that treatment with Akt inhibitors did not adversely affect hESC-derived CD34^+^ HPC viability, we measured cytotoxicity in HPCs treated with each inhibitor at 7 days post-treatment and did not observe changes in metabolic activity at the concentrations used in these studies (S1E and S2C Figs). To examine the role of Akt signaling at the time of reactivation, hESC-derived CD34^+^ HPCs were infected with TB40/E-GFP at a multiplicity of infection (MOI) of 2 for 48 hours, and viable, CD34^+^, GFP^+^ cells were sorted and seeded into long-term bone marrow culture (LTBMC) over stromal cell support to allow for the establishment of latent infection. After 12 days of LTBMC culture, HPCs were stimulated to differentiate and re-initiate virus replication by seeding onto monolayers of permissive fibroblasts in an extreme limiting dilution assay (ELDA) in cytokine-rich media supplemented with Afuresertib, BAY1125976 or DMSO. An equivalent number of cells were mechanically lysed and plated similarly to measure free infectious virus and serve as a pre-reactivation control [40,41]. As shown in Figs 1 and S3, we observed an increase in infectious centers when cells were treated with either Afuresertib (p = 0.0071) or BAY1125976 (p = 0.0103). The enhanced reactivation was not due to effects on HCMV replication in fibroblasts, as treatment of fibroblasts with either inhibitor does not alter HCMV replication kinetics compared to DMSO treatment (S1F–S1I and S2D–S2G Figs). These data support the hypothesis that Akt signaling restricts HCMV reactivation in hESC-derived CD34^+^ HPCs.

### HCMV miRNAs modulate Akt expression via multiple indirect mechanisms

**Fig 2:**
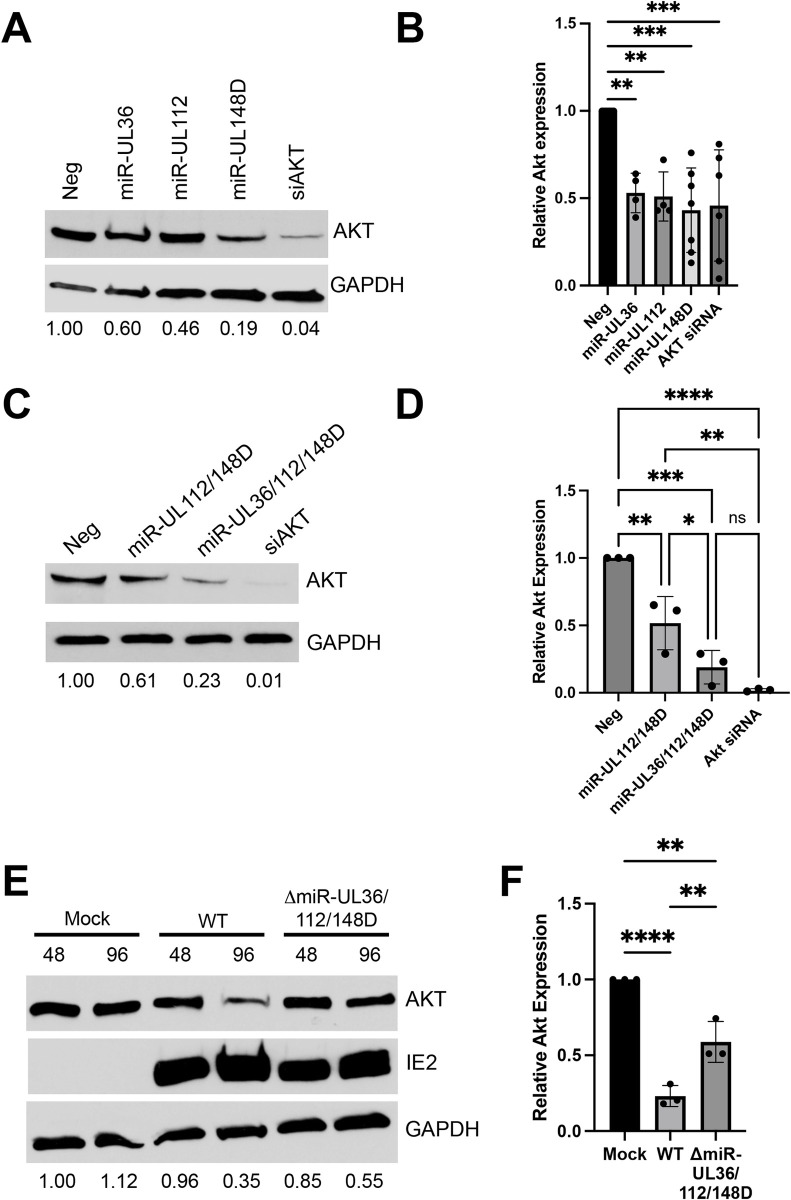
Akt is downregulated by HCMV miR-UL36, miR-UL112, and miR-UL148D. (A-D) NHDFs were transfected with double-stranded miRNA mimics, negative control (Neg), or siRNA. Lysates were harvested 72hr post-transfection and immunoblotted for Akt and GAPDH (loading control). Quantification from one representative blot shows relative expression levels of Akt compared to Neg (normalized to GAPDH). (B, D) Quantification of (A, C), respectively, from at least three separate experiments (*p<0.05, **p<0.005, ***p<0.0005, ****p<0.0001 [one-way ANOVA]). (E) NHDFs were infected at an MOI of 3 PFU/cell with WT HCMV (TB40/E-GFP), a mutant lacking miR-UL36, miR-UL112, and miR-UL148D expression (ΔmiR-UL36/112/148D), or uninfected (Mock). Lysates were harvested 48 or 96 hpi and immunoblotted for Akt, HCMV IE2, and GAPDH. Quantification shows relative expression levels of Akt compared to Neg (normalized to GAPDH). (F) Quantification of (E) at 96 hpi from three separate experiments (**p<0.005, ****p<0.0001 [one-way ANOVA with Tukey’s multiple comparison test]).

Since our data indicates that Akt activity is repressive to HCMV reactivation in hESC-derived CD34^+^ HPCs, we hypothesized that HCMV has evolved mechanisms to inhibit Akt at this critical time in the virus lifecycle. We have previously shown that HCMV-encoded miRNAs play significant roles in CD34^+^ HPC infection [10,11,42] and can disrupt signaling pathways necessary for efficient reactivation from latency [12]. Knowing this, we sought to determine whether any of the HCMV-encoded miRNAs affect Akt expression. To this end, we transfected HCMV or negative control miRNA mimics into normal human dermal fibroblasts (NHDFs) and assessed Akt expression levels. Western blot analysis showed that miR-UL36, miR-UL112, and miR-UL148D each reduced endogenous levels of Akt ~50% compared to negative control miRNA (Fig 2A and 2B), and co-transfection of all three miRNAs decreased Akt levels by approximately 75% (Fig 2C and 2D). Interestingly, transfection of only miR-UL112 and miR-UL148D did not reduce Akt levels as efficiently as when miR-UL36 was included, suggesting that all three miRNAs contribute to maximal reduction in Akt expression. We next assessed whether these HCMV miRNAs affect Akt expression during HCMV lytic infection. To this end, we used bacterial artificial chromosome (BAC) recombineering to generate a mutant virus lacking expression of all three HCMV miRNAs in HCMV TB40/E-GFP (ΔmiR-UL36/112/148D). We infected NHDFs with wild-type (WT) HCMV or ΔmiR-UL36/112/148D at an MOI of 3, and whole cell lysates were harvested at 48- and 96-hours post-infection (hpi). Western blot analysis demonstrated a decrease in Akt expression at 96 hpi in WT-infected cells. However, infection with ΔmiR-UL36/112/148D resulted in enhanced Akt expression compared to WT at this time point (Fig 2E and 2F). The decrease in Akt expression late during WT infection contrasts previous studies, which examined Akt expression levels 12–48 hours after infection and did not observe changes in total Akt levels [29,30,43–45]. The ΔmiR-UL36/112/148D virus grew with WT kinetics (S4 Fig), suggesting that the effects on Akt expression are not due to changes in replication kinetics of the virus. These observations contrast the effects of Akt dysregulation on HCMV replication in other artificial systems [29], but miRNA regulation of Akt happens late in infection when viral progeny are already being produced. Taken together, the data indicate that miR-UL36, miR-UL112, and miR-UL148D contribute to reducing Akt expression during HCMV lytic infection in fibroblasts.

**Fig 3:**
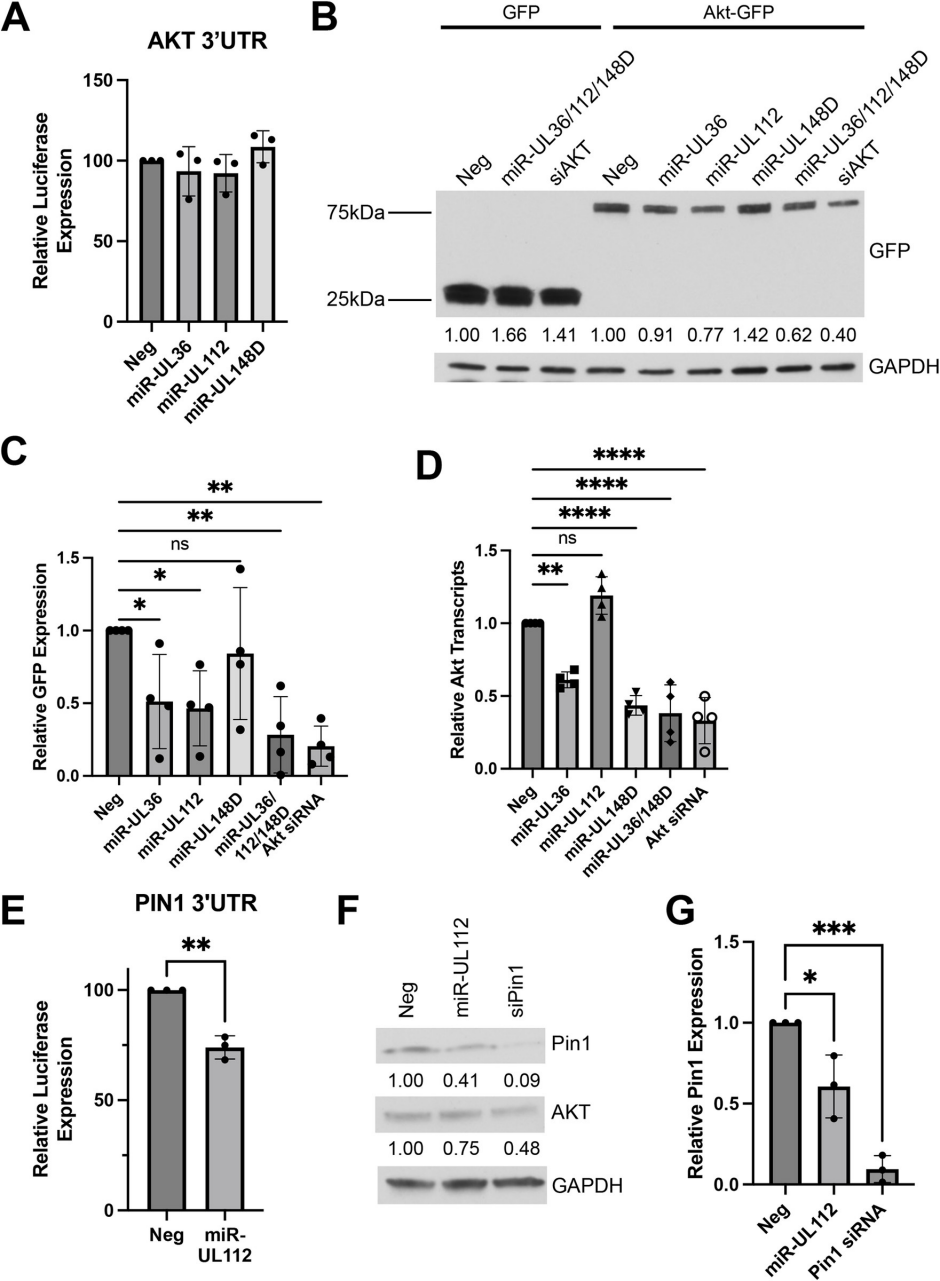
HCMV miRNAs inhibit Akt through non-canonical mechanisms. (A, E) A dual luciferase reporter containing the 3’UTR of Akt (A) or Pin1 (E) was cotransfected into HEK293T cells along with miRNA mimics. Luciferase expression was assessed 24hrs post-transfection. The relative expression is shown as a percentage of Neg. Error bars represent the standard deviation from three separate experiments (**p<0.005 by unpaired t-test). (B) HEK293T cells were co-transfected with GFP or Akt-GFP and miRNA mimics or siRNA. Lysates were harvested 24hr post-transfection and immunoblotted for GFP and GAPDH. Quantification from one representative blot shows relative expression levels of Akt-GFP compared to Neg (normalized to GAPDH). (C) Quantification of (B) from four separate experiments (*p<0.05, **p<0.005 [one-way ANOVA with Tukey’s multiple comparison test]). (D) NHDF cells were transfected with miRNA mimics, negative control (Neg), or siRNA. RNA has harvested 72hr post-transfection, and quantitative RT-PCR for Akt was performed. Expression levels were normalized to 18S and compared to Neg (**p<0.005, ****p<0.0001 [one-way ANOVA with Tukey’s multiple comparison test]). (F) NHDF were transfected with miRNA mimics or siRNA. Lysates were harvested 72hr post-transfection and immunoblotted for Pin1, Akt, and GAPDH. Quantification from one representative blot shows relative expression levels of Akt compared to Neg (normalized to GAPDH). (G) Quantification of (F) from three separate experiments (*p<0.05, ***p<0.0005 [one-way ANOVA with Tukey’s multiple comparison test]).

Canonical miRNA targeting occurs via complementarity between the 3’ untranslated region (UTR) of a target mRNA and the seed sequence of the miRNA [46]. To test whether miR-UL36, miR-UL112, or miR-UL148D directly target the Akt 3’ UTR, we co-transfected miRNA mimics (or negative control) into HEK293T cells along with a luciferase reporter plasmid containing the 3’ UTR of Akt. To our surprise, neither miR-UL36, miR-UL112, nor miR-UL148D affected luciferase expression compared to negative control mimic (Fig 3A), suggesting these miRNAs do not affect Akt expression by directly targeting the Akt 3’UTR. To further investigate HCMV miRNA targeting of Akt, we transfected a plasmid containing only the Akt protein coding sequence (CDS) tagged with GFP, along with HCMV miRNA mimics, Akt siRNA, or negative control mimics and whole cell lysates were harvested 24 hours post-transfection. Western blot analysis showed that miR-UL36 and miR-UL112 reduced levels of Akt-GFP compared to negative control (Fig 3B and 3C), suggesting that these miRNAs affect Akt protein expression in a mechanism independent of UTR targeting. Finally, we assessed the effects of HCMV miRNAs on Akt transcript levels. NHDFs transfected with miR-UL36 or miR-UL148D, but not miR-UL112, significantly showed significantly reduced Akt transcript levels compared to negative control mimic (Fig 3D). This suggests that, in HEK293 and/or fibroblasts, miR-UL36 and miR-UL148D affect Akt protein levels by causing reduced mRNA levels, while miR-UL112 acts at the level of protein expression.

Since miR-UL112 reduces Akt protein expression but does not directly target the Akt 3’ UTR or affect Akt transcript levels, we hypothesized that miR-UL112 may indirectly modulate expression by targeting a regulator of Akt. One such protein is Pin1, an isomerase that promotes the stability of Akt [47]. miR-UL112 decreased expression of luciferase driven by the Pin1 3’ UTR compared to negative control conditions (Fig 3E), suggesting miR-UL112 directly targets Pin1. Moreover, transfection of miR-UL112 reduced endogenous levels of Pin1 in HEK293T cells (Fig 3F and 3G) and expression of miR-UL112 or Pin1 knockdown also reduced endogenous levels of Akt (Fig 3F), consistent with a model whereby miR-UL112 destabilizes Akt protein by reducing expression of Pin1. Taken together, our data suggest that HCMV miRNAs inhibit Akt expression via mechanisms independent of conventional 3’ UTR targeting that include affecting Akt mRNA expression and targeting regulators of Akt stability.

### miR-UL36, miR-UL112, and miR-UL148D alter Akt signaling in fibroblasts

**Fig 4:**
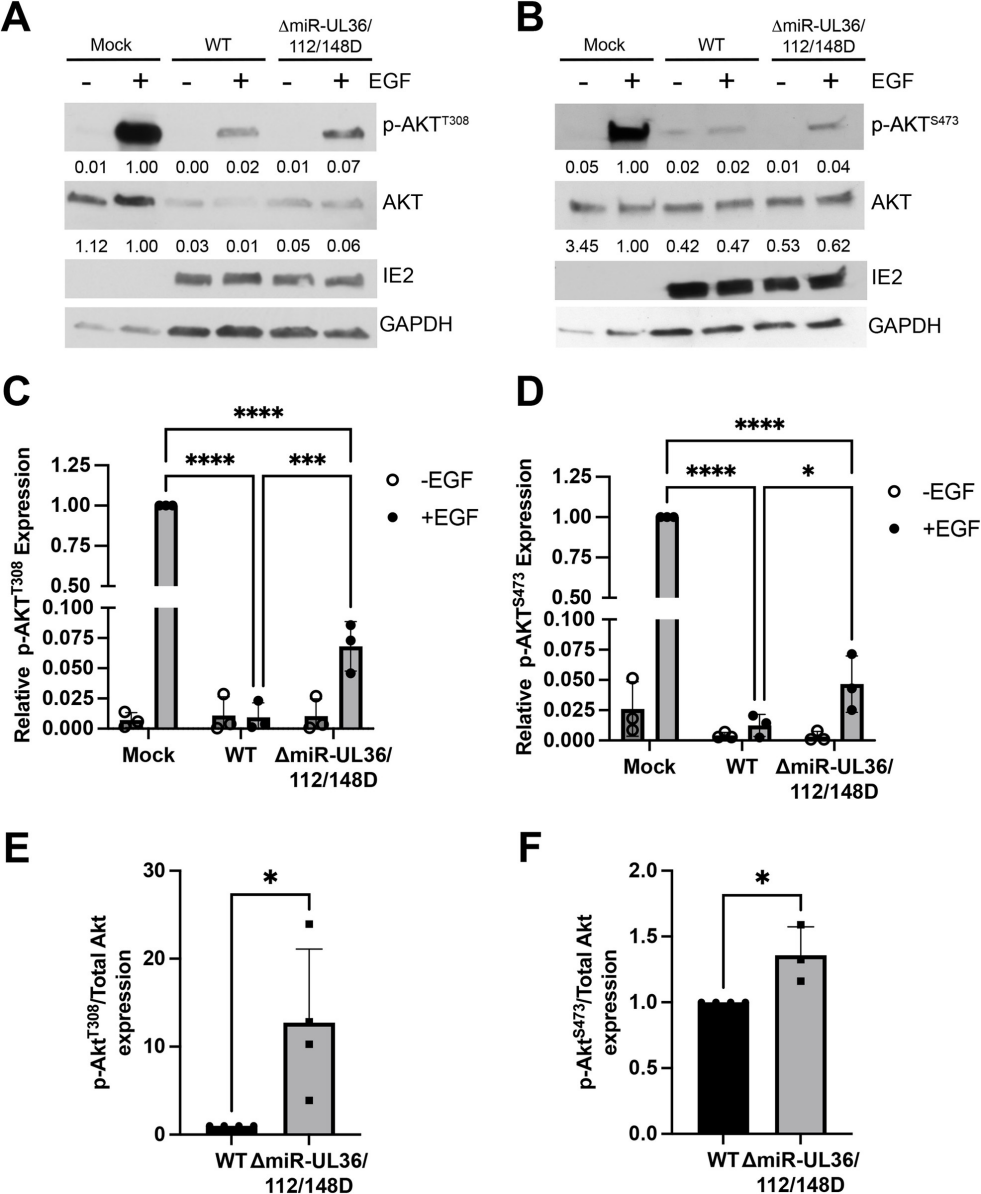
HMCV miRNAs inhibit Akt phosphorylation in lytic infection. (A,B) NHDF were infected at an MOI of 3 with WT, ΔmiR-UL36/112/148D, or Mock infected for 48hr, serum starved overnight, and then stimulated +/-EGF for 15 minutes. Lysates were harvested and immunoblotted for Akt phosphorylated at T308 (A) or S473 (B) as well as total Akt, HCMV IE2, and GAPDH. Quantification from one representative blot shows relative expression levels of p-Akt and total Akt compared to Mock (normalized to GAPDH). (C, D) Quantification of p-Akt levels from (A, B), respectively, from three separate experiments (comparing +EGF conditions *p<0.05, ***p<0.0005, ****p<0.0001 [two-way ANOVA with Tukey’s multiple comparison test]). (E, F) Ratio of p-Akt to total Akt levels (normalized to GAPDH and relative to WT) comparing WT and ΔmiR-UL36/112/148D from (A, B), respectively, from four separate experiments, normalized to WT (*p<0.05 [unpaired t-test]).

**Fig 5:**
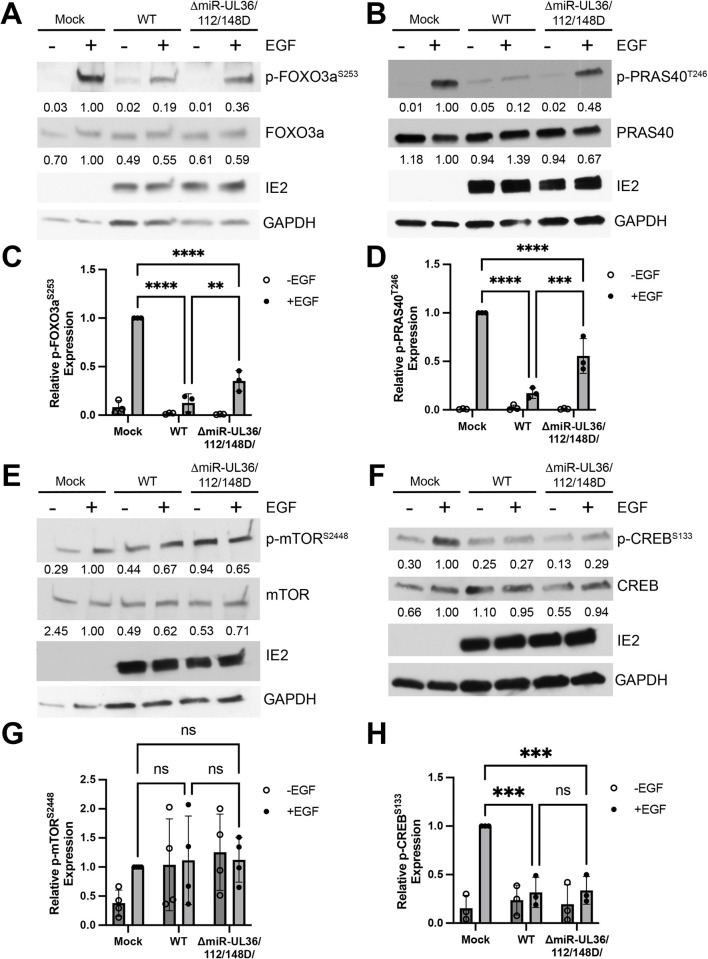
HCMV miRNAs affect signaling downstream of Akt during lytic infection. (A, B, E, F) NHDF were infected at an MOI of 3 with WT, ΔmiR-UL36/112/148D, or Mock infected for 48hr, serum starved overnight, and then stimulated +/- EGF for 15 minutes. Lysates were harvested and immunoblotted for indicated phosphorylated and total proteins as well as HCMV IE2 and GAPDH. Quantification from one representative blot shows relative expression levels of p-protein and total protein compared to Mock (normalized to GAPDH). (C, D, G, H) Quantification of (A, B, E, F), respectively, from three separate experiments (comparing +EGF conditions, **p<0.005, ***p<0.0005, ****p<0.0001 [two-way ANOVA with Tukey’s multiple comparison test]).

Knowing that Akt protein levels are reduced by HCMV miRNAs, we asked whether the miRNAs also alter signaling downstream of Akt during lytic infection. To test this, NHDFs were infected at an MOI of 3 with WT HCMV, ΔmiR-UL36/112/148D, or Mock infected for 48 hr. Cells were serum starved overnight, followed by treatment +/- EGF for 15 minutes in order to stimulate Akt phosphorylation, and lysates were collected and analyzed for phosphorylated and total protein levels. Western blot analysis showed that treatment of uninfected cells with EGF robustly induced phosphorylation of Akt at residues Threonine 308 (Fig 4A and 4C) and Serine 473 (Fig 4B and 4D). Cells infected with WT HCMV showed greatly reduced p-AKT levels in addition to reduced total Akt expression compared to Mock, consistent with previous reports that Akt activation is inhibited during HCMV infection [29,30,43–45]. However, cells infected with ΔmiR-UL36/112/148D consistently showed increased levels of p-Akt at both phosphorylation sites (Fig 4A–4D), along with enhanced Akt protein expression. Interestingly, we observed a striking increase in the proportion of p-Akt compared to total Akt in ΔmiR-UL36/112/148D-infected cells (Fig 4E and 4F), suggesting that the miRNAs alter Akt activation in addition to Akt expression. We next asked if signaling downstream of Akt is also affected by changes in total and p-Akt levels. Similar to the results with p-Akt, ΔmiR-UL36/112/148D infection resulted in enhanced phosphorylation of several Akt substrates compared to WT HCMV infection, including FOXO3a (Fig 5A and 5C) and PRAS40 (Fig 5B and 5D). However, compared to WT HCMV infection, no significant difference was observed in phosphorylated levels of Akt substrates mTOR (Fig 5E and 5G), CREB (Fig 5F and 5H), or P70S6K (S5 Fig), likely as these components are direct or indirect targets of other viral gene products [31–33,43]. Together, these data demonstrate that miR-UL36, miR-UL112, and miR-UL148D reduce total and p-Akt levels in infected fibroblasts, which is necessary to affect downstream AKT signaling pathways not co-opted by other virus-mediated processes.

### HCMV miRNAs affect FOXO3a nuclear localization and function in fibroblasts

**Fig 6:**
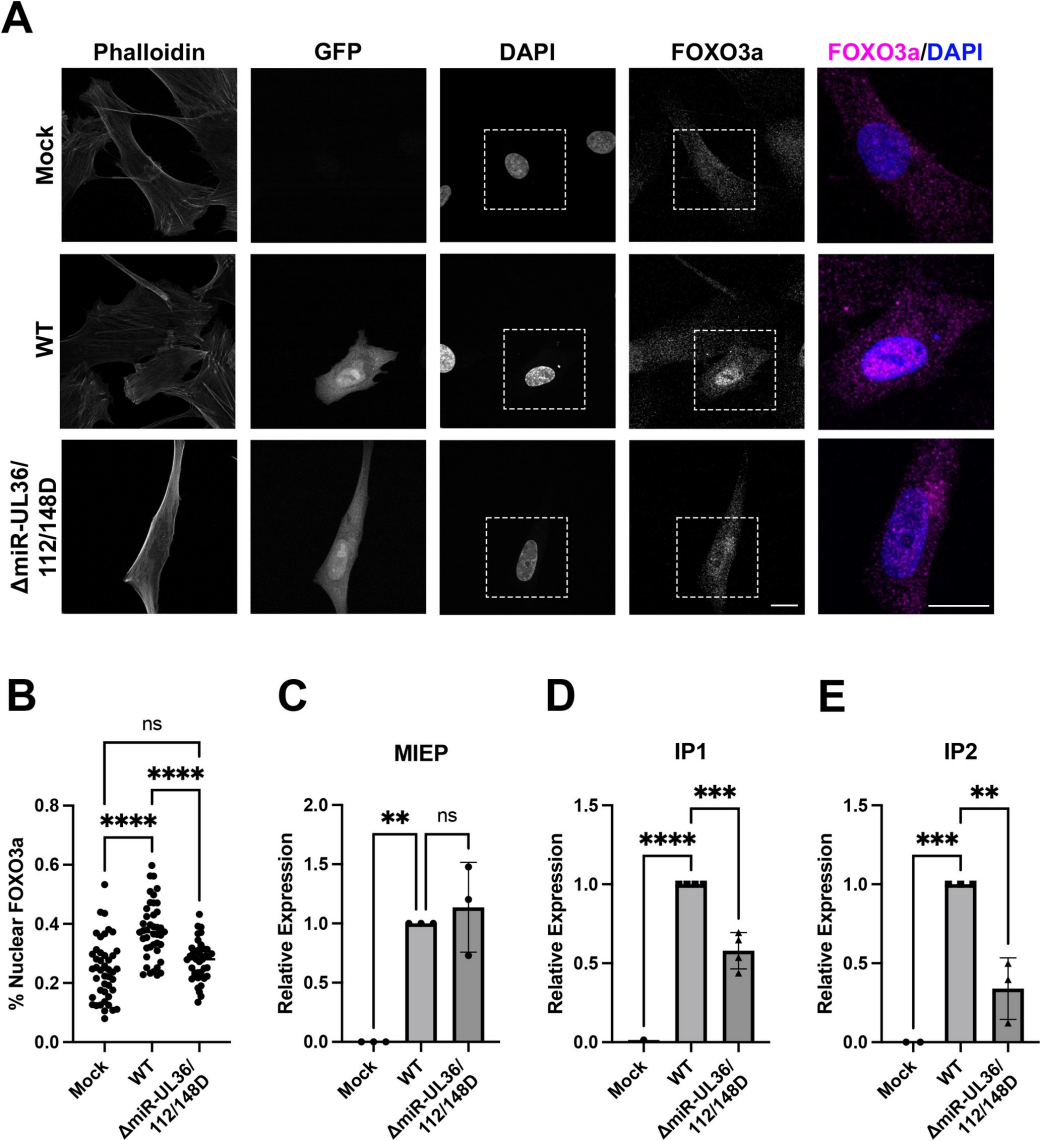
HCMV miRNAs promote FOXO3a nuclear localization and induction of MIE transcripts during lytic infection. (A, B) NHDF were plated on coverslips and infected at an MOI of 0.1 with WT, ΔmiR-UL36/112/148D, or Mock infected. Cells were fixed 72 hpi and stained for actin (phalloidin), FOXO3a, and nuclei (DAPI). (A) Representative images are shown. White dotted lines indicate region of interest used for magnified images (right panel). Right panel images show overlay of FOXO3a (magenta) and nuclei (blue). Scale bar, 20μM. (B) Image J software was used to quantify the average intensity of FOXO3a in the nucleus and the entire cell and graphed as a percentage of nuclear FOXO3a levels normalized to the whole cell. Individual values for 38–44 cells from each condition from three separate experiments are shown (****p<0.0001 [one-way ANOVA with Tukey’s multiple comparison test]). (C-E) NHDF were infected with WT, ΔmiR-UL36/112/148D, or Mock infected for 72hr and RNA has harvested. Quantitative RT-PCR was performed using specific primers for MIEP (C), IP1 (D), or IP2 (E). Expression levels were normalized to GAPDH and compared to WT from at least three separate experiments (**p<0.003, ***p<0.0007, ****p<0.0001 [one-way ANOVA with Tukey’s multiple comparison test]).

FOXO3a is an important effector regulated by Akt signaling during HCMV infection [36, 48]. FOXO3a in its active, unphosphorylated form localizes to the nucleus and acts as a transcription factor to regulate cellular homeostasis, stress responses, and apoptosis [34, 49]. Our data suggest that HCMV miR-UL36, miR-UL112, and miR-UL148D together reduce FOXO3a phosphorylation in fibroblasts (Fig 5A and 5C). Thus, we hypothesized that HCMV miRNAs promote nuclear translocation of FOXO3a. To test this, NHDFs were infected at an MOI of 0.1 with WT HCMV, ΔmiR-UL36/112/148D, or Mock infected for 72hr and immunostained for FOXO3a, DAPI, and actin (phalloidin). Cells infected with WT HCMV showed an ~64% increase in FOXO3a nuclear localization compared to mock (Fig 6A and 6B), consistent with a major population of unphosphorylated, active FOXO3a in WT-infected cells. In cells infected with the ΔmiR-UL36/112/148D mutant, FOXO3a localized throughout the cell (Fig 6A) and nuclear FOXO3a levels were not significantly different from mock infection (Fig 6B).

Recent work has identified two alternative intronic promoters (iP1 and iP2) that play an important role in stimulating IE gene expression during reactivation from latency in bone marrow-derived CD34^+^ HPCs [36,50]. Furthermore, FOXO3a binding sites in intron A of the major immediate early (MIE) locus contribute to expression from these promoters [36]. Given our observations that HCMV miR-UL36, miR-UL112, and miR-UL148D promote FOXO3a activation during HCMV lytic infection (Figs 5A, 5C, 6A and 6B), we hypothesized that a downstream consequence would be the induction of iP1 and iP2 transcripts. To test this, we infected NHDF at an MOI of 3 with WT HCMV, ΔmiR-UL36/112/148D, or Mock infected, harvested RNA 72 hours later and performed qPCR for MIEP-, iP1-, or iP2-derived transcripts. While infection with ΔmiR-UL36/112/148D showed similar levels of MIEP transcripts to WT infection (Fig 6C), ΔmiR-UL36/112/148D-infected cells produced significantly lower amounts (p = 0.0006 and p = 0.0022, respectively) of transcripts derived from iP1 and iP2 promoters (Fig 6D and 6E, respectively). Together, these data suggest that inhibition of Akt in fibroblasts by HCMV miRNAs results in reduced FOXO3a phosphorylation, increased translocation to the nucleus, and IE gene transcription from promoters that are important for reactivation from latency.

### HCMV miR-UL36, miR-UL112, and miR-UL148D promote reactivation from latency *in vitro* and *in vivo*

**Fig 7:**
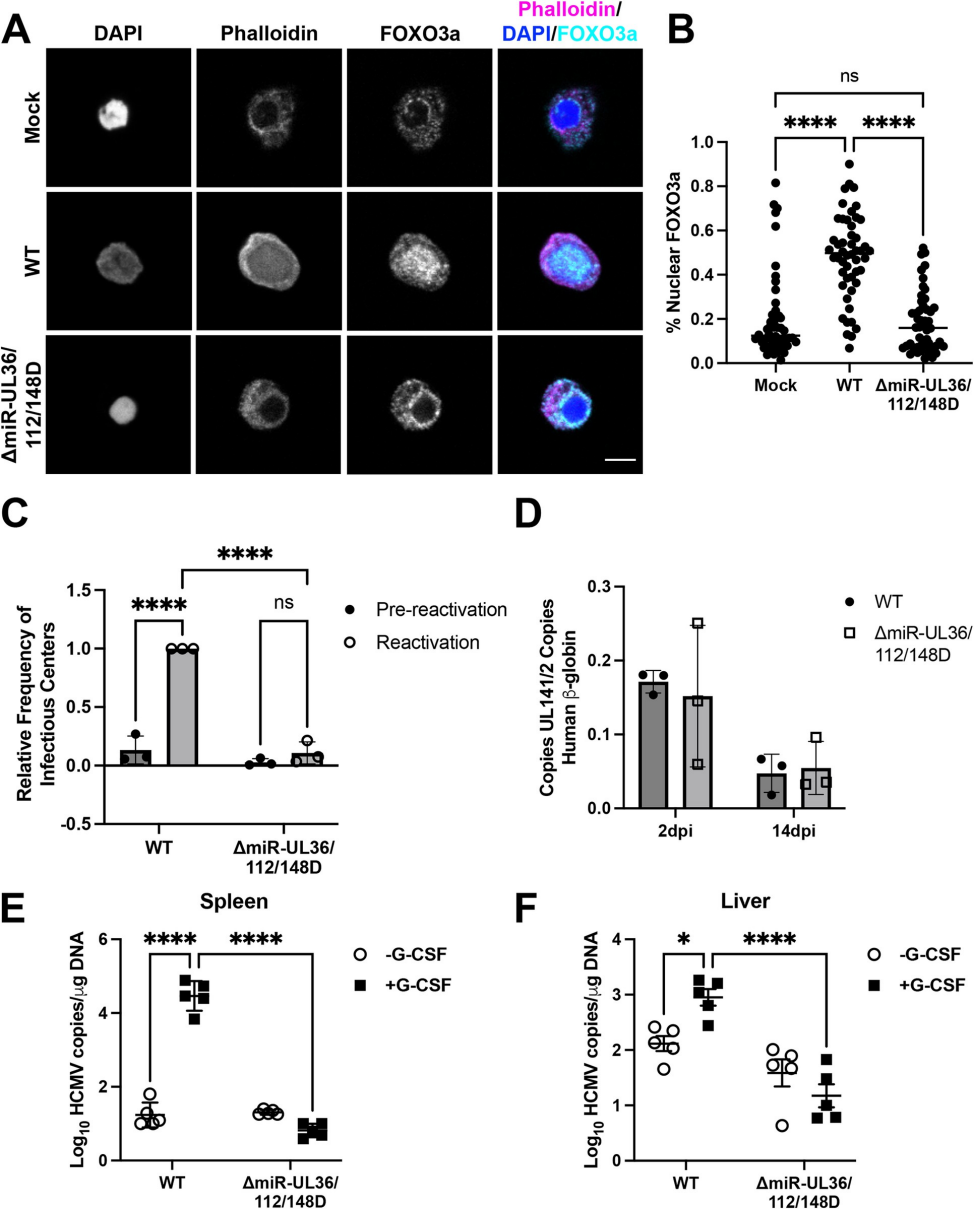
miR-UL36, miR-UL112, and miR-UL148D are important for HCMV reactivation from latency *in vitro* and *in vivo*. (A, B) hESC-derived CD34^+^ HPCs were infected at an MOI of 2 with WT, ΔmiR-UL36/112/148D, or Mock for 48hr and then sorted by FACS for viable, CD34^+^, GFP^+^ cells. (A) HPCs were maintained in SFEM hematopoietic cell media overnight, and then cells were fixed and seeded onto gelatin-coated coverslips and stained for actin (phalloidin), FOXO3a, and nuclei (DAPI). Representative images are shown. Right panel shows overlay of Phalloidin (magenta), FOXO3a (cyan) and nuclei (blue). Scale bar, 5 μM. (B) Image J software was used to quantify the average intensity of FOXO3a in the nucleus and the entire cell and graphed as a percentage of nuclear FOXO3a levels normalized to the whole cell. Individual values for 50 cells from each condition from three separate experiments are shown (****p<0.0001 [one-way ANOVA with Tukey’s multiple comparison test]). (C, D) Infected HPCs were maintained in LTBMC culture medium in transwells over stromal cells for 12 days to establish latency. (C) Latently-infected cells were co-cultured with NHDFs in cytokine-rich media in an extreme limiting dilution assay (ELDA) to measure viral reactivation. An equal number of cells were mechanically disrupted and seeded in parallel to measure infectious virus present in the latency culture (pre-reactivation). At 21 days post-plating, the number of GFP^+^ wells were scored and the frequency of infectious center production was determined by ELDA software. Reactivation is shown as the relative frequency of infectious center compared to WT-infected cells from three separate experiments (****p<0.0001 [one-way ANOVA with Tukey’s multiple comparison test]). (D) Total genomic DNA was isolated from HPCs at 2 days post infection (dpi) (post-sort) or 14 dpi (latency day 12), and quantitative real-time PCR was used to quantify the ratio of viral genomes (copies of HCMV UL141) to cellular genomes (per two copies of human β-globin). Error bars represent standard deviation from triplicate samples. Data shown are representative of three independent experiments. (E, F) Sub-lethally irradiated NOD-*scid* IL2Rγ_c_^null^ mice were engrafted with CD34^+^ HPCs (huNSG) and subsequently injected with human fibroblasts previously infected with HCMV WT or ΔmiR-UL36/112/148D. At 4 weeks post-infection, viral reactivation was triggered by treating latently infect HCMV WT and ΔmiR-UL36/112/148D (n = 5) with G-CSF and AMD-3100. At 1 week post-treatment, mice were euthanized, and tissues were harvested. Total genomic DNA was isolated from spleen tissue (E) or liver tissue (F), and HCMV genomes were quantified using qPCR with primers and probes specific for the UL141 gene (*p<0.05, ***p<0.0005, ****p<0.0001 [two-way ANOVA followed by Bonferroni’s multiple comparison test]).

Given the role of miR-UL36, miR-UL112, and miR-UL148D in regulating FOXO3a activity and cellular localization in fibroblasts, we sought to determine whether these miRNAs also affect Akt signaling in CD34^+^ HPCs. To this end, we infected hESC-derived CD34^+^ HPCs at an MOI of 2 with WT, ΔmiR-UL36/112/148D, or Mock infected for 48 hours, and viable, CD34^+^, GFP^+^ cells were sorted and cultured an additional 24 hours before seeding onto gelatin-coated coverslips and immunostained for FOXO3a, DAPI, and actin (phalloidin). Similar to results from infected NHDFs, WT infection resulted in a distinctly nuclear localization of FOXO3a (Fig 7A), indicative of FOXO3a activation early during HCMV infection of hESC-derived HPCs. In contrast, Mock infection as well as infection with ΔmiR-UL36/112/148D showed a distinct cytoplasmic FOXO3a localization (Fig 7A) and significantly lower nuclear FOXO3a levels compared to WT infection (Fig 7B). Thus, our data indicates that the HCMV miRNAs that affect Akt levels also regulate FOXO3a translocation in hematopoietic cells.

Since we have shown that Akt signaling impairs virus reactivation (Figs 1 and S3) and miR-UL36, miR-UL112, and miR-UL148D reduce Akt protein levels (Figs 2, 3 and 4) and IE transcripts from promoters shown to be important for reactivation in bone marrow-derived CD34^+^ HPCs (Fig 6D and 6E), as well as FOXO3a translocation in hESC-derived CD34^+^ HPCs (Fig 7A and 7B), we next assessed the ability of the ΔmiR-UL36/112/148D mutant to reactivate from latency in CD34^+^ HPCs. Infection of hESC-derived CD34^+^ HPCs with the ΔmiR-UL36/112/148D mutant infected cells showed a significant decrease (p<0.0001) in the frequency of infectious center production compared to WT infected cells (Figs 7C and S6). Moreover, the frequency of reactivation in ΔmiR-UL36/112/148D-infected cells was not significantly different than the pre-reactivation control, suggesting that when HCMV is lacking miR-UL36, miR-UL112, and miR-UL148D, the virus is unable to reactivate from latency in hESC-derived CD34^+^ HPCs efficiently. HCMV genome levels were similar for both viruses at the beginning and end of latency (Fig 7D), indicating that the reactivation defect of the ΔmiR-UL36/112/148D mutant is not due to differences in initial binding and entry or a loss of viral genomes or genome-containing cells during latency.

To further support the findings that a ΔmiR-UL36/112/148D virus is unable to reactivate from latency, we assessed reactivation of this virus in an *in vivo* humanized mouse model [51–59]. CD34^+^ HPCs were engrafted into NOD-*scid*IL2Rγ_c_^null^ mice (huNSG) followed by infection with WT HCMV or ΔmiR-UL36/112/148D. After viral latency was established, HCMV reactivation was stimulated by treatment of mice with granulocyte colony-stimulating factor (G-CSF) and AMD3100 which results in virus dissemination into tissues through infected macrophages [54]. WT-infected animals showed significantly increased DNA copy numbers in both spleen (Fig 7E) (p<0.0001) and liver (Fig 7F) (p = 0.031) tissues of huNSG mice following reactivation stimulus. Critically, no change in DNA copy number was observed in ΔmiR-UL36/112/148D-infected mice triggered to reactivate, demonstrating that miR-UL36, miR-UL112, and miR-UL148D are also important for reactivation from latency *in vivo*.

### Akt inhibition by HCMV miRNAs contributes to reactivation from latency in hESC-derived CD34^+^ HPCs

**Fig 8:**
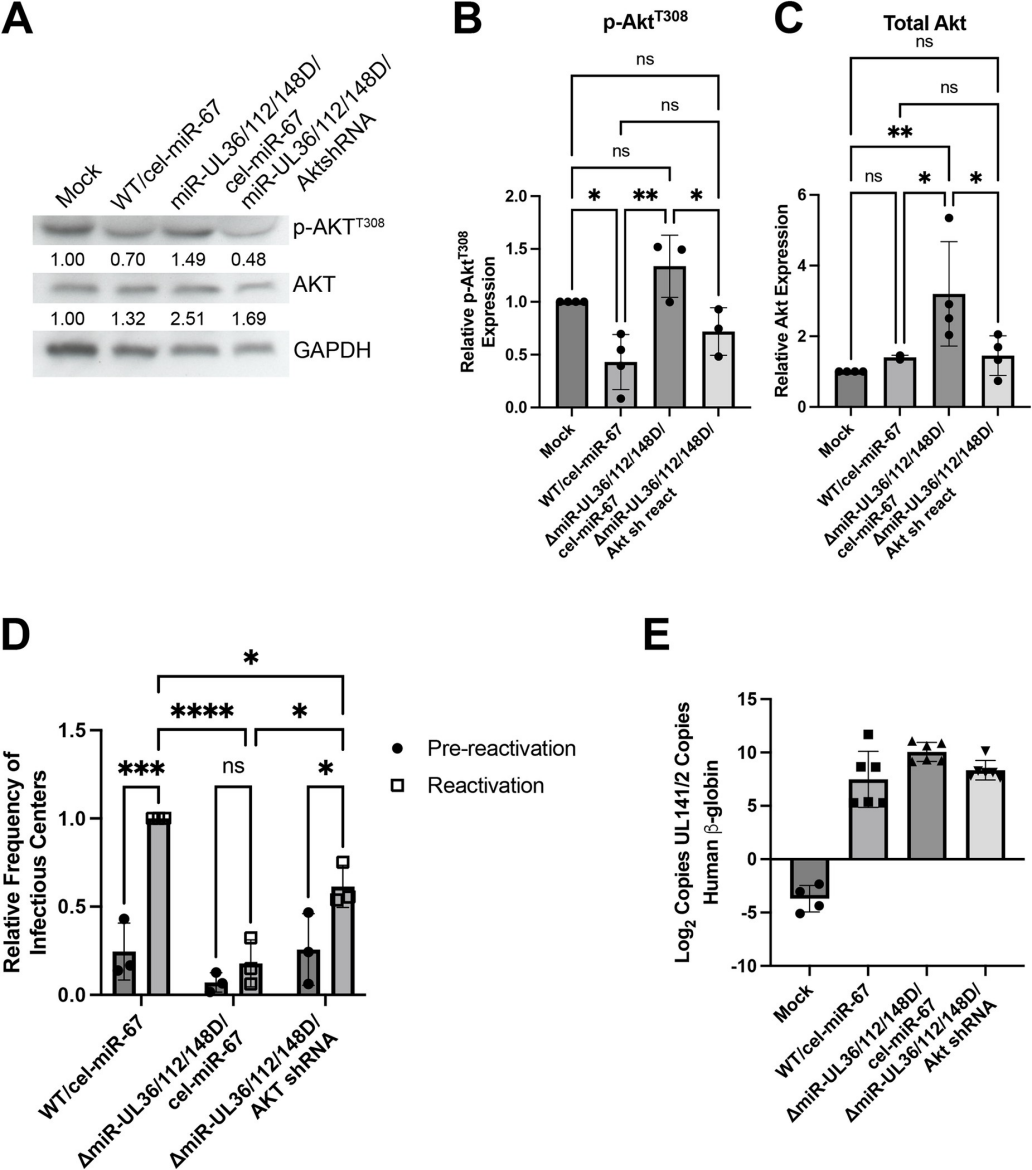
HCMV miRNA regulation of Akt contributes to reactivation from latency in hESC-derived CD34^+^ HPCs. hESC-derived CD34^+^ HPCs were infected at an MOI of 2 with WT HCMV expressing cel-miR-67 (WT/cel-miR-67), ΔmiR-UL36/112/148D/cel-miR-67, UL36/112/148D/Akt shRNA, or Mock infected for 48hr and then sorted by FACS for viable, CD34^+^, GFP^+^ cells. (A) HPCs were cultured overnight and then lysates were harvested and immunoblotted for p-Akt (T308), Akt, and GAPDH. Quantification from one representative experiment shows relative expression levels of p-Akt and total Akt compared to Mock (normalized to GAPDH). (B, C) Quantification of p-Akt (B) or total Akt (C) levels from (A), from at least three separate experiments (*p<0.05, **p<0.005 [one-way ANOVA with Tukey’s multiple comparison test]). (D) Sorted cells were used in latency and reactivation assays as described in Fig 7. Reactivation is shown as the relative frequency of infectious centers compared to WT-infected cells from three independent experiments (*p<0.05, ***p<0.0005, ****p<0.0001 [two-way ANOVA with Tukey’s multiple comparison test]). (E) Total genomic DNA was isolated from HPCs 14 dpi, and quantitative real-time PCR was used to quantify the ratio of viral genomes as described in Fig 7. Error bars represent standard deviation from triplicate samples. Data shown are from two independent experiments.

While we have shown that miR-UL36, miR-UL112-3p and miR-UL148D-3p each independently regulate Akt expression (Fig 2), each miRNA targets several additional proteins [8,60–71], many of which have not been investigated in the context of latency. In order to determine if the reactivation defect of the ΔmiR-UL36/112/148D mutant is due to its inability to regulate Akt protein levels specifically, we generated a mutant HCMV lacking expression of miR-UL36, miR-UL112, and miR-UL148D as well as expressing an shRNA targeting Akt in the 3’UTR of HCMV UL22A (ΔmiR-UL36/112/148D/Akt shRNA), a highly abundant transcript expressed during latency. As a control, we generated a virus expressing a non-targeting *Caenorhabditis elegans* miRNA, cel-miR-67, in this same region in both WT HCMV (WT/cel-miR-67) and the ΔmiR-UL36/112/148D mutant (ΔmiR-UL36/112/148D/cel-miR-67) (S7A Fig). To confirm that these viruses modulate Akt levels we quantified Akt transcripts in NHDFs infected with WT/cel-miR-67, ΔmiR-UL36/112/148D/cel-miR-67, ΔmiR-UL36/112/148D/Akt shRNA, or Mock infected. Akt transcript levels were decreased in WT/cel-miR-67-infected cells at 96hpi compared to mock. NHDFs infected with ΔmiR-UL36/112/148D/cel-miR-67 showed significantly higher (p = 0.044) Akt transcript levels than WT, consistent with the effects of ΔmiR-UL36/112/148D virus on Akt protein levels. As predicted, the ΔmiR-UL36/112/148D/Akt shRNA virus showed decreased Akt transcripts compared to the ΔmiR-UL36/112/148D/cel-miR-67 virus (S7B Fig), effectively complementing the lack of Akt regulation of the ΔmiR-UL36/112/148D/cel-miR-67 mutant. As expected, neither ΔmiR-UL36/112/148D/cel-miR-67 nor ΔmiR-UL36/112/148D/Akt shRNA exhibited a significant lytic replication defect compared to WT/cel-miR-67 (S7C–S7F Fig). Importantly, hESC-derived CD34^+^ HPCs infected with ΔmiR-UL36/112/148D/cel-miR-67 showed increased levels of p-Akt (Fig 8A and 8B) and total Akt (Fig 8A and 8C) compared to WT/cel-miR-67- and ΔmiR-UL36/112/148D/Akt shRNA-infected cells. Furthermore, transfection of primary human monocytes with miR-UL36, miR-UL112 and miR-UL148D also reduced Akt phosphorylation in response to EGF or HCMV binding (S8 Fig). Combined with the results of Fig 7A and 7B, these findings suggest that, like in fibroblasts, miR-UL36, miR-UL112, and miR-UL148D inhibit Akt expression and signaling in myeloid cells, including hESC-derived CD34^+^ HPCs.

We next assessed the ability of the ΔmiR-UL36/112/148D/Akt shRNA virus to reactivate from latency in hESC-derived CD34^+^ HPCs. WT/cel-miR-67 virus is able to establish latency and reactivate similarly to WT virus (S9A and S9B Fig). Furthermore, the ΔmiR-UL36/112/148D/cel-miR-67 virus exhibited a reactivation defect (Figs 8D and S9C) similar to the ΔmiR-UL36/112/148D virus (Fig 7C). Moreover, genomes were maintained in ΔmiR-UL36/112/148D/cel-miR-67-infected cells compared to infection with WT/cel-miR-67 (Fig 8E), similar to infection with ΔmiR-UL36/112/148D (Fig 7D). Finally, infection with the ΔmiR-UL36/112/148D/Akt shRNA virus demonstrated an enhanced frequency of infectious centers compared to ΔmiR-UL36/112/148D/cel-miR-67 (Fig 8D), but no change in genome copy number (Fig 8E), suggesting that inhibiting Akt in the context of infection with the ΔmiR-UL36/112/148D mutant is able to partially complement the reactivation defect observed when these miRNAs are lacking during infection. Taken together, these data suggest that miR-UL36, miR-UL112, and miR-UL148D promote HCMV reactivation from latency in hESC-derived CD34^+^ HPCs via a mechanism at least partially dependent on reducing Akt expression.

## Discussion

In this study, we provide the first mechanistic evidence of how HCMV regulates Akt signaling at the time of reactivation from latency. We show that three miRNAs encoded by HCMV—miR-UL36, miR-UL112, and miR-UL148D—coordinately inhibit Akt expression and alter downstream Akt signaling during lytic and latent infection. By modulating Akt protein levels, these HCMV miRNAs prevent the phosphorylation and inactivation of FOXO3a, thereby promoting nuclear localization and inducing expression of MIE transcripts containing FOXO3a binding sites during infection of fibroblasts. Importantly, we show that an HCMV mutant lacking expression of the three miRNAs fails to reactivate from latency both *in vitro* and *in vivo*, yet expression of an Akt shRNA can partially complement the reactivation defect, indicating that reducing Akt expression is one mechanism used by HCMV miRNAs to promote reactivation.

Our data clearly demonstrate that miR-UL36, miR-UL112, and miR-UL148D affect Akt protein levels both when ectopically expressed (Fig 2A–2D) and during HCMV infection (Figs 2E, 2F, 8A and 8C). Surprisingly, none of these miRNAs target the 3’ UTR of Akt in a canonical manner (Fig 3A). Each miRNA reduces Akt levels independently, and potentially through different mechanisms. miR-UL36 reduces both RNA (Fig 3D) and protein levels (Fig 2A–2D) and affects expression of Akt-GFP from a plasmid lacking the 3’ and 5’ UTRs (Fig 3B and 3C), suggesting miR-UL36 may target the CDS of Akt. Binding of miRNAs to the CDS commonly occurs with extensive base pairing at the 3’ end of the mature miRNA [72]. Indeed, we observed a region of complementarity in the Akt CDS with miR-UL36. However, CDS targeting typically results in translational repression without affecting mRNA levels [73,74], although this is not always the case [72,75]. Therefore, we cannot yet determine whether miR-UL36 directly or indirectly affects Akt. miR-UL148D, on the other hand, reduces Akt transcript (Fig 3D) and protein levels (Fig 2A–2D), but does not target the 3’UTR (Fig 3A) or the coding region (Fig 3B and 3C). We also did not find any predicted target sites for miR-UL148D in the 5’ UTR of Akt. Taken together, these data point to a role for miR-UL148D indirectly affecting Akt mRNA expression. The IER5 transcription factor was identified as a miR-UL148D target [63], but further study is needed to determine if transcription factor targeting by miR-UL148D is responsible for the effects on Akt transcription. Lastly, miR-UL112 expression decreases Akt protein (Fig 2A–2D) but not RNA levels (Fig 3D) and does not target the 3’ UTR (Fig 3A). Here we identify the isomerase Pin1 as a target of miR-UL112. Pin1 aids in stabilizing active Akt and preventing its degradation [47]. Our data show that miR-UL112 targets the 3’ UTR of Pin1 (Fig 3E) and ectopic expression of miR-UL112 decreases both Pin1 and Akt levels (Fig 3F and 3G). Thus, our data suggest that one mechanism by which HCMV miRNAs reduce Akt levels is by targeting Pin1 to induce Akt degradation. These findings highlight the myriad ways that viral miRNAs can affect expression of a single protein to elicit a phenotypic effect.

Akt phosphorylation is induced upon HCMV entry into fibroblasts [29,30] and monocytes [28] but in fibroblasts is downregulated within 12 hrs post-infection [29,30]. Consistent with this, we observed decreased Akt phosphorylation in response to EGF stimulation during infection of fibroblasts and hESC-derived CD34^+^ HPCs with WT HCMV. Interestingly, total Akt levels did not appear to be affected during WT infection of hESC-derived CD34^+^ HPCs (Fig 8A and 8C), in contrast to observations during lytic infection of fibroblasts (Fig 5A–5D). The reason for this discrepancy is currently unclear, however, during infection with the ΔmiR-UL36/112/148D mutant, total and p-Akt levels were higher than during WT infection (Figs 4 and 8A–8C), suggesting that these miRNAs act to reduce activation as well as expression of Akt. Downregulation of total Akt levels has not been noted in previous studies [29,30,43–45], but this study examined later timepoints (72–96 hpi) than previously reported. Hence, miRNA downregulation of total Akt levels is a second, late mechanism used by HCMV to alter this signaling pathway. Since removing these miRNAs from HCMV only partially restored p-Akt levels compared to Mock infection, this suggests that other HCMV factors inhibit Akt activation during infection. Indeed, HCMV UL38 contributes to inhibition of Akt phosphorylation via a negative feedback loop involving mTORC1 and IRS1 [30]. Given that infection with the ΔmiR-UL36/112/148D mutant shows increased p-Akt levels despite UL38 expression suggests that these miRNAs also play a direct role in regulating Akt signaling apart from regulating total Akt levels (Figs 4E, 4F, 8A and 8B). Interestingly, we observed a greater increase in phosphorylation at T308 than S473 during lytic infection (Fig 4E and 4F) which suggests that HCMV miRNAs preferentially regulate pathways leading to T308 phosphorylation mediated by PDK1. We did not observe any changes in expression of PDK1, a kinase known to phosphorylate Akt at Thr308 [76], or phosphorylation of mTOR (Fig 5E and 5G), which is part of the mTORC2 complex that phosphorylates Akt at Ser473 [77] and thus further study is needed to identify other potential regulators of Akt phosphorylation targeted by the miRNAs. Intriguingly, during lytic infection miR-UL36, miR-UL112, and miR-UL148D only affect activation of a subset of the tested Akt effectors, including FOXO3a, PRAS40, and Akt itself, but not other effectors like CREB, mTOR, and P70S6K. UL38 bypasses the need for Akt activity to maintain protein translation, making mTOR and P70S6K immune to the downstream effects of miRNA-mediated Akt regulation. HCMV regulation of CREB phosphorylation is less well understood, but CREB binding sites in the MIEP are necessary for efficient reactivation from latency [78]. Our data indicate that CREB phosphorylation is regulated by a mechanism that is protected from miRNA-regulated, Akt-mediated phosphorylation (Fig 5F and 5H). While the current study cannot distinguish between modulation of targets downstream of Akt directly due to Akt downregulation versus, or in addition to, other indirect effects, the fact that downstream effectors controlled by other viral proteins are immune to miRNA-mediated modulation suggests that miRNA regulation of Akt likely plays a role in their activity. Regulation of Akt via three HCMV miRNAs is also necessary to modulate FOXO3a activity and localization in fibroblasts and hESC-derived CD34^+^ HPCs (Figs 5A, 5B, 6A, 6B, 7A and 7B). Interestingly, FOXO3a is a target of miR-US5-1 and miR-UL112, whose downregulation is important for inhibiting apoptosis early after infection of CD34^+^ HPCs [42]. We did not observe a change in total FOXO3a expression during infection with ΔmiR-UL36/112/148D (Fig 5A), suggesting miR-UL112 alone is insufficient to functionally affect FOXO3 levels during infection. However, our current study suggests that miR-US5-1 and miR-UL112 downregulation of FOXO3a, along with the effectors of Akt signaling, may work together to block virus replication during latency establishment. Taken together, our current study, along with previously published work, highlights the growing evidence that HCMV miRNAs can target multiple components of a signaling pathway to alter downstream functional outcomes [11,65,69,70,79]. Clearly, HCMV regulates Akt activation and signaling in myriad ways, and our data points to HCMV miRNAs as contributors to this regulation, most appreciably during reactivation from latency in hESC-derived CD34^+^ HPCs.

**Fig 9:**
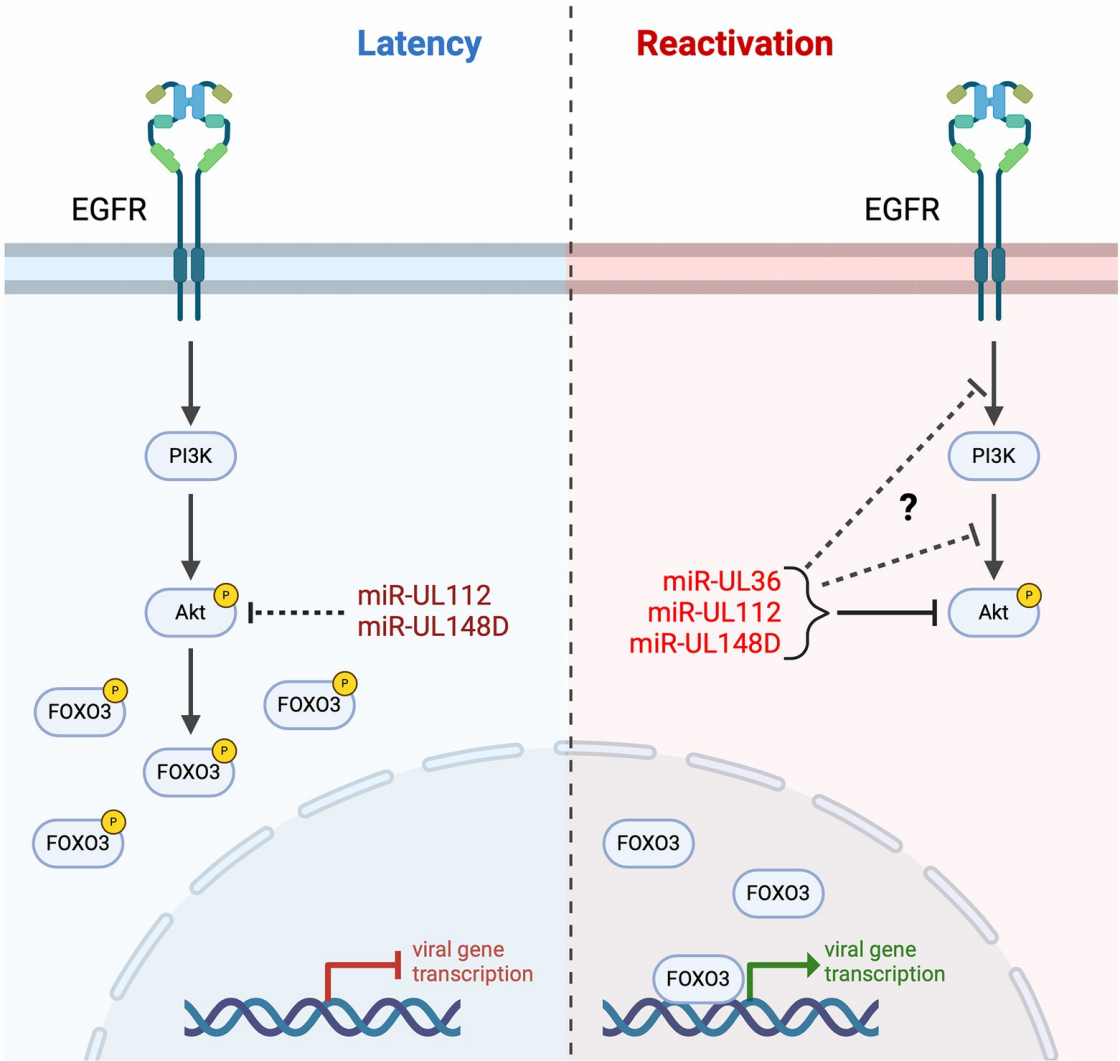
Model of Akt regulation in HCMV latency and reactivation. Left, EGFR and Akt signaling is required for latency. Active Akt promotes phosphorylation and inhibition of FOXO3a, thereby limiting viral gene transcription. Right, during reactivation, miR-UL36, miR-UL112, and miR-UL148D are expressed, which reduce Akt expression and activation. These miRNAs in turn promote active FOXO3a nuclear translocation and transcription of MIE genes.

In bone marrow- and hESC-derived CD34^+^ HPCs, EGFR/Akt signaling is required for establishment and/or maintenance of latency, as treatment of HPCs with Akt inhibitors results in enhanced virus replication compared to untreated conditions (S1A Fig; [35]). We hypothesize that the intensity of Akt signaling acts as a switch between latency and reactivation; EGFR/Akt signaling is involved in reducing virus replication during latency establishment via an unknown mechanism, but Akt signaling must be attenuated at the time of reactivation in order to stimulate replication. In support of this model, treatment of HPCs with two different Akt inhibitors when cells are stimulated to reactivate results in enhanced reactivation in WT-infected cells (Figs 1 and S3), suggesting that Akt activity restricts some aspects of the reactivation process. miRNA regulation of Akt levels contributes to the process of reactivation, and this is dependent on expression of multiple HCMV miRNAs. While miR-UL112 and miR-UL148D are expressed during latency, miR-UL36 is not detected (S10 Fig; [12]). Furthermore, expression of miR-UL112 and miR-UL148D only partially reduce Akt levels compared to expression of all three miRNAs (Fig 2C and 2D). Therefore, we hypothesize that during latency miR-UL112 and miR-UL148D are unable to influence Akt activity enough to tip the balance towards reactivation. However, the additional expression of miR-UL36 during the early stages of reactivation reduces Akt signaling enough to have a phenotypic effect on signaling and to stimulate virus replication (Fig 9). In agreement with this, infection with the ΔmiR-UL36/112/148D mutant, which results in increased Akt activity compared to WT-infected cells, exhibits a reactivation defect *in vitro* and *in vivo* (Fig 7), but this defect can be partially overcome by expression of an Akt shRNA (Fig 8). Importantly, these findings are the first to show that HCMV miRNAs have a phenotypic function *in vivo*, further underscoring the importance of virally encoded miRNAs to the HCMV lifecycle. Our data also highlight the complexity of miRNA regulation of the processes of latency and reactivation. Previous work shows that a miR-UL148D mutant virus has a replicative phenotype in CD34^+^ HPCs [63] while a miR-UL112 mutant showed no specific defect in latency or reactivation in CD14^+^ monocytes [80]. When mutations to miR-UL112, miR-UL148D and miR-UL36 are combined, the resulting mutant is unable to reactivate, indicating that the replicative phenotype of the miR-UL148D mutant can be overcome with the loss of additional miRNAs. Unravelling the important function(s) of each miRNA will help to understand the phenotypes of each individual and combination mutant. Together, our data for the first time describes a miRNA-mediated mechanism for Akt inhibition during HCMV reactivation and adds to the growing evidence of the importance of modulating Akt activity in CD34^+^ HPCs.

HCMV reactivation from latency is a multi-step, multi-component process which depends on re-expression of viral genes that are largely silenced during latency, including IE1 and IE2. While IE1 and IE2 transcripts are mostly driven by the MIEP during lytic infection, recent work has uncovered that expression of genes from the MIE locus are driven by two alternative promoters, iP1 and iP2, in bone marrow-derived CD34^+^ HPCs. In CD14^+^ monocytes, IE expression is driven by the MIEP and iP2, while fully differentiated macrophages and dendritic cells solely utilize the MIEP, suggesting differentiation-dependent utilization of promoters from the MIEP region in myeloid cells [81]. Deletion of the transcriptional start sites for iP1 and/or iP2 results in a virus that is unable to reactivate from latency in bone marrow-derived CD34^+^ HPCs [50]. Moreover, FOXO3a binding sites in this region are important for iP1- and iP2-driven transcript expression and reactivation in bone marrow-derived CD34^+^ HPCs [36]. In lytic infection, phosphorylated Akt restricts HCMV replication in a mechanism dependent on inactivating FOXO3a. Expressing a constitutively active Akt impaired IE transcript accumulation, including iP1 and iP2-driven transcripts, resulting in a defect in viral DNA synthesis. However, artificially inducing FOXO3a nuclear localization in ARPE epithelial cells was able to overcome the inhibition of IE transcription induced by constitutive Akt activity [29]. The mechanisms underlying the regulation of Akt and FOXO3a signaling during HCMV lytic and latent infection have not yet been fully elucidated, but our data demonstrate a role for miR-UL36, miR-UL112, and miR-UL148D in reducing Akt expression and signaling in hESC-derived CD34^+^ HPCs and thereby promoting FOXO3a activation and nuclear translocation. Furthermore, infection of fibroblasts with the ΔmiR-UL36/112/148D virus resulted in reduced iP1- and iP2-driven transcript accumulation (Fig 6D and 6E), but no change in MIEP expression (Fig 6C), supporting a link between Akt levels and iP1 and iP2 transactivation. The ΔmiR-UL36/112/148D mutant is unable to reactivate from latency both *in vitro* and *in vivo*, similar to preventing FOXO3a binding to iP1 and iP2 regions of the viral genome. Critically, introducing an Akt shRNA into the miRNA mutant virus partially restored the ability of the virus to reactivate, underscoring the important role for miRNA-mediated attenuation of Akt signaling during reactivation.

Akt is a central kinase in the cell involved in numerous essential cellular functions, and so reduction of Akt protein levels by HCMV miRNAs may also affect additional processes that contribute to reactivation from latency, such as modulating myeloid differentiation. HCMV infection of monocytes induces Akt activation and drives differentiation into macrophages [27,28,82–86], and so further study is needed to assess the effects of reducing Akt signaling on myeloid differentiation. Intriguingly, recent findings suggest that Akt activity is attenuated very early after infection of monocytes via US28, which is necessary to limit virus replication [28]. These findings suggest that HCMV regulation of Akt activity following direct infection of monocytes may be substantially different than during reactivation and myeloid differentiation from HPCs. Nevertheless, our findings support previously published works in lytic and latent infection establishing a role for regulation of Akt signaling as well as the requirement of Akt attenuation to allow for FOXO3a activation and IE gene expression, modelling for the first time a mechanism employed by HCMV miRNAs to regulate Akt during reactivation.

## Materials and methods

### Ethics statement

For collection of human monocytes, the study protocol was approved by the Institutional Review Board at Louisiana State University Health Sciences Center (Protocol #H99-064). We collected signed informed consent forms from healthy donors aged 18 years or older. Animal experiments were approved by the Institutional Animal Care and Use Committee at Oregon Health & Science University (IACUC Protocol #0922). All animal experiments were carried out in strict accordance with the recommendations of the American Association for Accreditation of Laboratory Animal Care (AALAC).

### Cells and media

Feeder-free hESCs were obtained from WiCell (WA01-H1, hPCSC Reg identifier (ID) WAe001-A, NIH approval no. NIHhESC-10-0043). Cells were thawed and plated on Matrigel-coated six-well plates in complete mTeSR1 (Stem Cell Technologies). CD34^+^ HPCs were differentiated using a commercial feeder-free hematopoietic differentiation kit (STEMdiff Hematopoietic Kit, Stem Cell Technologies). HEK293 and adult normal human dermal fibroblasts (NHDF) were obtained from ATCC and cultured in Dulbecco’s modified Eagle’s medium (DMEM) supplemented with 10% heat-inactivated fetal bovine serum (FBS; Hyclone), 100 units/ml penicillin, 100 μg/ml streptomycin, and 100 μg/ml glutamine (Thermofisher). M2-10B4 and S1/S1 stromal cells were obtained from Stem Cell Technologies and maintained in DMEM with 10% FBS and penicillin, streptomycin, and glutamine as previously described [87]. Human monocytes were isolated from human peripheral blood obtained from healthy donors through venipuncture [88]. The blood was centrifuged using a Ficoll Histopaque 1077 gradient (Sigma-Aldrich, Inc., St. Louis, MO). Platelets were removed via multiple washes with isotonic saline (Braun Medical, Inc., Irvine, CA). Monocytes were then isolated from the total mononuclear cell population by centrifugation through a Percoll gradient (Pharmacia Biotech, Inc., Piscataway, NJ). The purified monocytes were resuspended in RPMI 1640 (Cellgro Technologies, Omaha, NE) with 1% human serum (Sigma-Aldrich). This study is approved by the Louisiana State University Health Sciences Center-Shreveport Institutional Review Board (approval #: H99-064) and all Health Insurance Portability and Accountability Act guidelines were followed. All cells were maintained at 37°C and 5% CO2.

### Viruses

Viruses used in this study include BAC-generated WT TB40/E expressing GFP from the SV40 promoter [89,90]. TB40/E mutant viruses containing point mutations in the pre-miRNA sequences for miR-UL36, miR-UL112 and miR-UL148D and viruses expressing either cel-miR-67 or an Akt shRNA in the 3’UTR of UL22A were generated by galactokinase (galK)- mediated recombination [90,91]. Briefly, the galK gene was inserted into the region of the pre-miRNA hairpin using homologous recombination (miR-UL36 galK F: GAAATAAGAAAAATCCACGCACGTTGAAAACACCTGGAAAGAACGTGCCCGAGCGAACGTCCTCTTTCCAGGTGTCCCTGTTGACAATTAATCATCGGCA, miR-UL36 galK R: GCTCCGT TCGCGCAACGCCCTGGGGCCCTTCGTGGGCAAGATGGGCACCGTCTGTTCGCAAGGTAAGCCCCACGCTCAGCAAAAGTTCGATTTATTCAAC, miR-UL12 galK F: CACAGCATGAACACCAGATGCTCCCGGCGCTCTGACAGCCTCCGGATCACATGGTTACTCAGCGTCTGCCAGCCTCCTGTTGACAATTAATCATCGGCA, miR-UL112 galK R: CCTCGGGTTGCCTGGACGCCTGGGCGCGACGCGGCGTGCTGCTGCTCAACACCGTGTTCACCGTGGTGCACGGACTCAGCAAAAGTTCGATTTATTCAAC, miR-UL148D galK F: GAGGCAGAAGCTCGGTTCTCCAGGGACGACCGTCGATGCGTGGTAGGCGCCCTGTTGACAATTAATCATCGGCA, miR-UL148D galK R: AACTATCTGCAGAACACAAGGAAAAAGAAACACCAACCGAGGGTGGGTGGCTCAGCAAAAGTTCGATTTA), or the galK gene was inserted into the 3’UTR of pUL22A (F: AAGACTGATGAACACAAAGAAAATCAAGCCAAAGAAAATGAAAAGAAGATTCAGTAACAGCAGACCCCAAGGGTTAACGACCTGTTGACAATTAATCATC, R: AAAGAAAAAAGACCGGAGGCGGGGTGTTTTTAGAGCAAAACCTTACAGCTTTTTAATAAAAAACAAGGTAGTCAACATAACTCAGCAAAAGTTCGATTTA). In the second recombination step, galK is removed using oligos that encompass the pre-miRNA sequence containing point mutations in the hairpin (miR-UL36 F: CACCTGGAAAGAACGTGCCCGAGCGAACGTCCTCTTTCCAGGTGTCAAGTTGctCGTGGGGCTTACCTTGCGAACAGACGGTGCCCATCTTGCCCACGAA, miR-UL36 R: TTCGTGGGCAAGATGGGCACCGTCTGTTCGCAAGGTAAGCCCCACGAGCAACTTGACACCTGGAAAGAGGACGTTCGCTCGGGCACGTTCTTTCCAGGTG, miR-UL112 F: AGCCTCCGGATCACATGGTTACTCAGGCTCTGCCAGCCTAAATGCCGGTGAGAGCCCGGCTGTCCGTGCACCACGGTGAACACGGTGTTGAGCAGCAGCA, miR-UL112 R: TGCTGCTGCTCAACACCGTGTTCACCGTGGTGCACGGACAGCCGGGCTCTCACCGGCATTTAGGCTGGCAGACGCTGAGTAACCATGTGATCCGGAGGCT, miR-UL148D F: TGAGGTTGGGGCGGATAACGTGTTGCGGATCGTGGCGAGAACGTGGTGCTACCCTTCTTCACCGCCCCACCCACCCTCGGTTGGTGTTTCTTTTTCCTTG, miR-UL148D R: CAAGGAAAAAGAAACACCAACCGAGGGTGGGTGGGGCGGTGAAGAAGGGTAGCACCACGTTCTCGCCACGATCCGCAACACGTTATCCGCCCCAACCTCA) or inserting a cel-miR-67 (GCTGTTGACAGTGAGCGGCTACTCTTTCTAGGAGGTTGTGATAGTGAAGCCACAGATGTATCACAACCTCCTAGAAAGAGTAGATGCCTACTGCCTCGGA) or Akt shRNA sequence (TGCTGTTGACAGTGAGCGCGCGTGACCATGAACGAGTTTTAGTGAAGCCACAGATGTAAAACTCGTTCATGGTCACGCATGCCTACTGCCTCGGA). All virus stocks were propagated and titered on NHDFs using standard techniques. To assess growth kinetics, NHDFs were infected at a MOI of 3 for single-step growth curves or a MOI of 0.01 for multi-step growth curves for 2 hr. Cell-associated and supernatant virus was harvested at multiple time points post-infection. Titers were determined by plaque assay on NHDFs.

### Reagents

The 3’UTR of human Akt or Pin1 was amplified by PCR from fibroblast genomic DNA and cloned downstream of the *Renilla* luciferase gene in the psiCHECK-2 dual reporter construct (Promega) by XhoI (Akt) or SpeI (Pin1) and NotI restriction sites using the following primer pairs: Akt-GCGGCTCGAGCACACCACCTGACCAAGAT and CGCCGCGGCCGCGAAAAGCAACTTTTATTGAAGAATTTGGAG, Pin1- GCGCACTAGTGCAGAAGCCATTTGAAGACGC and GCGCGCGGCCGCGCAGACAGTGGTTCTGG. siGENOME RISC-free control siRNA (Neg; Dharmacon), Akt siRNA (s659; Thermofisher), and Pin1 siRNA (s10546, Thermofisher) were used in transfection experiments. Double stranded miRNA mimics were custom designed and synthesized by Integrated DNA Technologies. pEGFP-Akt1 (WT) was a gift from Thomas Leonard and Ivan Yudushkin (Addgene plasmid #86637; http://n2t.net/addgene:86637; RRID:Addgene_86637). The following commercial antibodies were used: Akt (C7H310, Cell Signaling), p-Akt S473 (9271, Cell Signaling), p-Akt T308 (13038, Cell Signaling), CREB (48H2, Cell Signaling), p-CREB S133 (87G3, Cell Signaling), FOXO3a (D19A7, Cell Signaling), p-FOXO3a S253 (D18H8, Cell Signaling), GFP (GF28R, Invitrogen), GAPDH (ab8245, Abcam), HCMV IE2 (MAB810, Sigma Aldrich), mTOR (7C10, Cell Signaling), p-mTOR S2448 (D9C2, Cell Signaling), P70S6K (PA5-17883, Cell Signaling), p-P70S6K T389 (B2H9L2, Thermofisher), Pin1 (3722, Cell Signaling), Phalloidin-AlexaFluor 647 (sc-363797, Santa Cruz Biotechnology), PRAS40 (D23C7, Cell Signaling), p-PRAS40 T246 (D4D2, Cell Signaling), α-rabbit-AlexaFluor 555. Afuresertib was purchased from Selleckchem and BAY1125976 was purchased from MedChemExpress.

### Luciferase assays

HEK293T cells were seeded into 96 well plates and transfected with 100 ng of psiCHECK-2 vector and 100 fmol of negative control or miRNA mimic using Lipofectamine 2000 (Invitrogen). Twenty-four hours after transfection cells were harvested for luciferase assay using the Dual-Glo Reporter Assay Kit (Promega) according to the manufacturer’s instructions. Luminescence was detected using a Veritas microplate luminometer (Turner Biosystems). All experiments were performed in triplicate and presented as mean +/- standard deviation.

### Transfection of monocytes

Briefly, monocytes were cultured in suspension in RPMI 1640 (Cellgro) with 1% human serum (Sigma-Aldrich) [88]. The human serum was removed for transfection, and then the monocytes (3 × 10^6^) were resuspended in 100 μl of room temperature nucleofection solution (human monocyte Nucleofector kit; Amaxa Biosystems, Cologne, Germany) containing a negative control small interfering RNA (siRNA) (scrambled control), a mixture of UL36+UL112+UL148D miRNAs, or an Akt siRNA (Ambion, Thermo Fisher Scientific, Waltham, MA). Following transfection, the cells were incubated in RPMI 1640 (Cellgro) supplemented with 1% human serum at 37°C with 5% CO^2^. After 48 hours, both mock and the transfected cells were serum starved for 12 hours, treated with human EGF (200 ng/mL), or infected with the HCMV TB40/E strain with a MOI of 5 for 30 minutes.

### Western blot analysis

Cells were harvested in protein lysis buffer (50mM Tris-HCl pH 8.0, 150mM NaCl, 1% NP40, and protease inhibitors), loading buffer (4X Laemmli Sample Buffer with 2-mercaptoethanol) was added, and lysates were incubated at 95°C for 5 min. Extracts were loaded onto 4–15% acrylamide gels (Biorad), transferred to Immobilon-P membranes (Millipore), and visualized with the specified antibodies. The relative intensity of bands detected by Western blotting was quantified using ImageJ software. For experiments using primary monocytes, cells were washed with PBS (Corning Inc, Corning, NY), then lysed in Pierce RIPA Buffer (Pierce Manufacturing, Appleton, WI) containing 1X Protease Inhibitor Cocktail (Thermo Fisher Scientific, Waltham, MA) and 1X Phosphatase Inhibitor Cocktail 1 (APExBio Technology, Houston, TX), collected whole cells and added vol/vol of Laemmli Sample Buffer (Bio-Rad, Hercules, CA) and lysates were incubated at 65°C for 10 min. The whole cell lysate was then loaded into a 12% sodium dodecyl sulfate (SDS)-polyacrylamide gel and transferred to polyvinylidene difluoride (PVDF) membranes (Millipore Sigma, Burlington, MA). The membranes were then blocked and incubated with primary antibodies phospho-AKT (Ser473), phospho-AKT (Thr308), pan-AKT, (Cell Signaling, Danvers, MA), or actin (Santa Cruz, Santa Cruz, CA). The immunoblots were subsequently incubated with IRDye 680RD and IRDye 800CW conjugated secondary antibodies (LI-COR, Lincoln, NE). After each antibody incubation step, the membranes were washed with PBS containing 0.5% Tween-20 (Thermo Fisher Scientific, Waltham, MA) 3 times for 10 min). Finally, membranes were scanned using a LI-COR Odyssey CLx Infrared Imaging System, and signals were quantified using LI-COR Image Studio software (Ver 5.2).

### Quantitative RT-PCR

Reverse transcription-PCR (RT-PCR) was used to quantitate cellular and viral RNA and viral miRNAs in infected NHDFs or CD34^+^ HPCs. Total RNA was isolated from infected cells using Trizol. cDNA was prepared using 1000ng of total RNA and random hexamer primers for cellular and viral RNAs, and using 100ng total RNA and miRNA hairpin-specific primers for viral miRNAs. Samples were incubated at 16°C for 30 minutes, 42°C for 30 minutes, and 85°C for 5 minutes. Real-time PCR (Taqman) was used to analyze cDNA levels in infected samples. An ABI StepOnePlus Real Time PCR machine was used with the following program for 40 cycles: 95°C for 15 sec and 60°C for 1 minute. Primer and probe sets for Akt (Hs00178289_m1) and 18S (Hs03928990_g1) were obtained from Thermo Fisher Scientific. Sequence-specific primer pairs for MIEP-, iP1-, and iP2-derived transcripts were used as previously described [50]. HCMV miRNA primers and probe sets for miR-UL36 (197212_mat), miR-UL112 (006621), and miR-UL148D (197215_mat) were obtained from Thermo Fisher Scientific. Relative expression was determined using the ΔΔCt method using 18S or GAPDH as the standard control with error bars representing the standard deviation from at least 3 experiments. For quantitation of miRNA copy number in latently infected HPCs, miRNA mimics representing the mature form of each miRNA were included in a serial dilution in independent RT reactions to determine absolute copy number.

### Microscopy

NHDFs were grown on 13mm glass coverslips and infected at an MOI of 0.1 with WT HCMV, ΔmiR-UL36/112/148D, or mock infected. 72 hpi, coverslips were washed with PBS and fixed with 4% paraformaldehyde in PBS. Cells were permeabilized with 0.25% Triton, blocked with normal goat serum, and stained with the indicated primary antibodies. Coverslips were then washed with PBS containing BSA and 0.1% Triton and incubated with the appropriate fluorophore-conjugated secondary antibodies. For experiments using CD34^+^ HPCs, cells were infected at an MOI of 2 with the indicated viruses for 48 hours and were sorted for CD34^+^, GFP^+^, viable cells. Sorted cells were cultured overnight in SFEM II hematopoietic media supplemented with 10% BIT serum replacement (Stem Cell Technologies), fixed with 4% paraformaldehyde in PBS and seeded onto gelatin-coated slides, permeabilized with 0.3% Triton and then stained with the indicated antibodies. Fluorescence was visualized using a LEICA Stellaris 8 microscope using the 63x objective with an NA of 1.4. The fluorophores were excited using 405nm and White Light Lasers. The signals were captured using Leica Stellaris 8 and the Leica Application Suite software. Images were exported as.tiff files and analyzed using ImageJ software.

### CD34^+^ HPC latency and reactivation assays

Differentiated hESCs were infected with the indicated viruses at an MOI of 2 for 48hr, or were left uninfected, in stem cell media (Iscove’s modified Dulbecco’s medium [IMDM] [Invitrogen] containing 10% BIT serum replacement [Stem Cell Technologies], penicillin/streptomycin, stem cell factor [SCF], FLT3 ligand [FLT3L], interleukin-3 [IL-3], interleukin-6 [IL-6] [all from PeproTech], 50uM 2-mercaptoethanol, and 20ng/ml low-density lipoproteins). Pure populations of viable, infected (GFP^+^) CD34^+^ HPCs were isolated by fluorescence-activated cell sorting (FACS) (BD FACSAria equipped with 488-, 633-, and 405-nm lasers and running FACSDiva software) and used in latency assays as previously described [87,92]. Briefly, cells were cultured in transwells above irradiated stromal cells (M2-10B4 and S1/S1) for 12 days to establish latency. Virus was reactivated by coculture with NHDF in RPMI medium containing 20% FBS, 1% P/S/G, and 15ng/ml each of G-CSF and GM-CSF in an extreme limiting dilution assay (ELDA). GFP^+^ wells were scored 3 weeks postplating and the frequency of infectious centers was using ELDA software [93]. In some experiments, Akt inhibitors 100nM Afuresertib or 50nM BAY1125976 (or DMSO control) was added to either the latency culture and replenished at 5 days of latency establishment, or inhibitors were added to the reactivation culture at the time of reactivation and replenished at 7 days post-reactivation.

### Engraftment and infection of humanized mice

NOD-*scid*IL2Rγ_c_^null^ mice were maintained in a pathogen-free facility at Oregon Health and Science University in accordance with procedures approved by the Institutional Animal Care and Use Committee. Both sexes of animals were used. Humanized mice were generated as previously described [54]. The animals (12–14 weeks post-engraftment) were treated with 1 ml of 4% Thioglycollate (Brewer’s Media, BD) by intraperitoneal (IP) injection to recruit monocyte/macrophages. At 24hr post-treatment, mice were infected with HCMV TB40/E-infected fibroblasts (approximately 10^5^ PFU of cell-associated virus per mouse) via IP injection. A control group of engrafted mice was mock infected using uninfected fibroblasts. Virus was reactivated as previously described [54].

### Quantitative PCR for viral genomes

DNA from CD34^+^ HPCs was extracted using the two-step TRIZOL (Thermofisher) method according to the manufacturer’s directions. Total DNA was analyzed in triplicate using TaqMan FastAdvanced PCR master mix (Applied Biosystems), and primer and probe for HCMV *UL141* and human β-globin as previously described [94]. Copy number was quantified using a standard curve generated from purified HCMV BAC DNA and human β-globin-containing plasmid DNA, and data were normalized assuming two copies of β-globin per cell.

### Statistical analysis

Statistical analysis was performed using GraphPad Prism software (v10) for comparison between groups using student’s t-test, one-way or two-way analysis of variance (ANOVA) with Tukey’s post-hoc test or Bonferroni’s multiple comparison test as indicated. Values are expressed as mean +/- standard deviation or standard error of the mean, as indicated in the figure legends. Significance is highlighted with p<0.05.

## Supporting information

S1 Fig Afuresertib inhibits latency establishment in hESC-derived CD34^+^ HPCs but does not affect cell viability or HCMV replication in fibroblasts. (A) hESC-derived CD34^+^ HPCs were infected with HCMV TB40/E-GFP at an MOI of 2 for 48hr and then sorted by FACS for viable, CD34^+^, GFP^+^ cells. Infected HPCs were maintained in LTBMC culture medium in transwells over stromal cells for 12 days to establish latency in the presence of Afuresertib (100 nM) or DMSO (control). Following the latency culture, cells were co-cultured in cytokine-rich media in an extreme limiting dilution assay (ELDA) to measure virus reactivation. An equal number of cells were mechanically disrupted and seeded in parallel to measure infectious virus present in the latency culture (pre-reactivation). At 21 days post-plating, the number of GFP^+^ wells were counted and the frequency of infectious center production was determined by ELDA software. Reactivation is shown as the relative frequency of infectious centers compared to DMSO control-treated cells from three independent experiments (***p<0.0005, ****p<0.0001 [two-way ANOVA with Tukey’s multiple comparison test]). (B-D) NHDFs were serum starved overnight and treated +/- 100nM Afuresertib. The next day, cells were stimulated +/- EGF for 15 minutes. Protein lysates were harvested and immunoblotted for p-Akt T308 (B), p-Akt S473 (C), or p-mTOR S2448 (D) as well as total Akt (B, C), total mTOR (D), and GAPDH. Quantification from one representative blot shows relative expression levels of p-AKT or p-mTOR compared to cells stimulated with EGF (normalized to GAPDH). (E) hESC-derived HPCs were incubated with the indicated concentrations of Afuresertib for 7 days. Cell viability was measured by WST-1 colorimetric assay (Roche) according to manufacturer’s instructions. Quantification shows absorbance at 450nm after background subtracting the value of media alone. Error bars represent standard deviation from triplicate samples from one representative experiment (*p<0.05 [one-way ANOVA with Tukey’s multiple comparison test]). (F-I) NHDFs were infected with WT TB40/E-GFP at an MOI of 3 for single-step (F, G) or an MOI of 0.01 for multistep (H, I) growth curves and treated +/- 100nM Afuresertib or DMSO (control). PFU/ml values were quantified in duplicate from samples collected at the indicated time points for cell-associated (F, H) or supernatant (G, I) virus.
(TIF)

S2 Fig BAY1125976 does not affect hESC-derived CD34^+^ HPC cell viability or HCMV replication in fibroblasts. (A, B) NHDFs were infected with WT TB40/E-GFP (or Mock infected) at an MOI of 3 for 8hr and then were serum starved overnight in the presence of increasing concentrations of BAY1125976. At 24 hpi, cells were stimulated with EGF for 15 minutes. Lysates were then harvested and immunoblotted for p-Akt T308 (A) or p-Akt S473 (B) as well as total Akt, IE2, and GAPDH. Quantification from one representative blot shows relative expression levels of p-AKT compared to Mock-infected cells stimulated with EGF (normalized to GAPDH). (C) hESC-derived CD34^+^ HPCs were incubated with the indicated concentrations of BAY1125976 for 7 days. Cell viability was measured by WST-1 colorimetric assay (Roche) according to manufacturer’s instructions. Quantification shows absorbance at 450nm after background subtracting the value of media alone. Error bars represent standard deviation from triplicate samples from one representative experiment (*p<0.05 [one-way ANOVA with Tukey’s multiple comparison test]). (D-G) NHDFs were infected with TB40/E-GFP at an MOI of 3 for single-step (D, E) or an MOI of 0.01 for multistep (F, G) growth curves and treated +/- 50nM BAY1125976. PFU/ml values were quantified in duplicate from samples collected at the indicated time points for cell-associated (D, F) or supernatant (E, G) virus.
(TIF)

S3 Fig Akt restricts HCMV reactivation in hESC-derived CD34^+^ HPCs. Individual experiments from Fig 1 show reactivation as the frequency of infectious centers for three replicate experiments for cells treated with Afuresertib (A) or BAY1125976 (B) during reactivation.
(TIF)

S4 Fig ΔmiR-UL36/112/148D virus does not have a growth defect during lytic infection. NHDFs were infected with WT TB40/E-GFP or ΔmiR-UL36/112/148D at an MOI of 3 for single-step (A, B) or an MOI of 0.01 for multistep (C, D) growth curves. PFU/ml values were quantified in duplicate from samples collected at the indicated time points for cell-associated (A, C) or supernatant (B, D) virus.
(TIF)

S5 Fig HCMV miR-UL36, miR-UL112, and miR-UL148D alter signaling downstream of Akt during lytic infection. (A) NHDF were infected at an MOI of 3 with WT, ΔmiR-UL36/112/148D, or Mock infected for 48hr, serum starved overnight, and then stimulated +/-EGF for 15 minutes. Lysates were then harvested and immunoblotted for phosphorylated and total P70S6K as well as HCMV IE2 and GAPDH. Quantification from one representative blot shows relative expression levels of p-P70S6K and total P70S6K compared to Mock (normalized to GAPDH). (C, D) Quantification of (A, B), respectively, from three separate experiments (comparing +EGF conditions, ****p<0.0001 [two-way ANOVA with Tukey’s multiple comparison test]).
(TIF)

S6 Fig miR-UL36, miR-UL112, and miR-UL148D are important for HCMV reactivation from latency *in vitro*. Individual experiments from Fig 7C show reactivation as the frequency of infectious centers for three replicate experiments.
(TIF)

S7 Fig Characterization of the ΔmiR-UL36/112/148D/Akt shRNA virus. (A) Schematic of cel-miR-67 and Akt shRNA-expressing viruses. From top to bottom: WT TB40/E-GFP, WT TB40/E-GFP expressing *C*. *elegans* miR-67 (cel-miR-67) from the 3’UTR of UL22A (WT/cel-miR-67), ΔmiR-UL36/112/148D expressing cel-miR-67 (ΔmiR-UL36/112/148D/cel-miR-67), or ΔmiR-UL36/112/148D expressing an Akt shRNA from this same region (ΔmiR-UL36/112/148D/Akt shRNA). (B) NHDFs were infected at an MOI of 3 with WT/cel-miR-67, ΔmiR-UL36/112/148D/cel-miR-67, ΔmiR-UL36/112/148D/Akt shRNA, or Mock infected for 24 or 96hr after which RNA was harvested. Quantitative RT-PCR was performed using specific primers for Akt. Expression levels were normalized to GAPDH and compared to Mock (*p<0.05, **p<0.005, ***p<0.0005, ****p<0.0001 [two-way ANOVA with Tukey’s multiple comparison test]). (C-F) NHDFs were infected with WT/cel-miR-67, ΔmiR-UL36/112/148D/cel-miR-67, or ΔmiR-UL36/112/148D/Akt shRNA at an MOI of 3 for single-step (C, D) or an MOI of 0.01 for multistep (E, F) growth curves. PFU/ml values were quantified in duplicate from samples collected at the indicated time points for cell-associated (C, E) or supernatant (D, F) virus.
(TIF)

S8 Fig Expression of miR-UL36, miR-UL112 and miR-UL148D diminishes phospho-Akt protein levels in primary human monocytes following treatment with EGF or HCMV binding. (A) Human monocytes were transfected with a negative control small interfering RNA (siRNA) (scrambled control; siNeg), a combination of miRNAs (miR-UL36+UL112+UL148D), or an siRNA targeting AKT (siAKT), and incubated for 48 hrs. 48 hrs after transfection, monocytes were infected with TB40/E at an MOI of 5 for 30 minutes or treated with hEGF for 30 minutes, and then protein lysates were harvested and immunoblotted for p-AKT (Ser473), p-AKT (Thr308), pan-AKT (PAN-AKT) and β-actin. (B) Relative band intensity was determined for pAKT (T308 and S473) compared to β-actin. A representative blot and band intensities are shown. 3 identical experiments with different human donors were performed and similar results were observed with all the donors.
(TIF)

S9 Fig HCMV miRNA regulation of Akt contributes to reactivation from latency in hESC-derived CD34^+^ HPCs. (A) hESC-derived CD34^+^ HPCs were infected with HCMV TB40/E-GFP or WT/cel-miR-67 at an MOI of 2 for 48hr and then sorted by FACS for viable, CD34^+^, GFP^+^ cells. Infected HPCs were maintained in LTBMC culture medium in transwells over stromal cells for 12 days to establish latency. Following the latency culture, cells were co-cultured in cytokine-rich media in an extreme limiting dilution assay (ELDA) to measure virus reactivation. An equal number of cells were mechanically disrupted and seeded in parallel to measure infectious virus present in the latency culture (pre-reactivation). At 21 days post-plating, the number of GFP^+^ wells were counted and the frequency of infectious center production was determined by ELDA software. Reactivation is shown as the relative frequency of infectious centers compared to DMSO control-treated cells from three independent experiments (**p<0.005 [two-way ANOVA with Tukey’s multiple comparison test]). (B) Individual experiments from S8A Fig show reactivation as the frequency of infectious centers for three replicate experiments. (C) Individual experiments from Fig 8D show reactivation as the frequency of infectious centers for three replicate experiments.
(TIF)

S10 Fig Expression of HCMV miR-UL36, miR-UL112, and miR-UL148D in latently infected hESC-derived CD34^+^ HPCs. hESC-derived CD34^+^ HPCs were infected at an MOI of 2 for 48 hours, then FACS-isolated for viable, CD34^+^, GFP^+^ HPCs. Sorted cells were cultured under latency conditions for 12 days to establish latency and HCMV miRNA levels were detected in 10ng RNA from infected cells by stem-loop qRT-PCR.
(TIF)

S1 Data Raw immunoblot data. Raw immunoblot data associated with main and supplemental figures.
(PDF)

S2 Data Master raw data file for the manuscript. Raw data associated with main and supplemental figures.
(XLSX)
