# Supplementary figures and images for: Viral microRNA regulation of Akt is necessary for reactivation of Human Cytomegalovirus from latency in CD34+ hematopoietic progenitor cells and humanized mice
Source: PLoS Pathog. 2024 Dec 11;20(12):e1012285. doi: 10.1371/journal.ppat.1012285 (PMC11666035; doi:10.1371/journal.ppat.1012285)

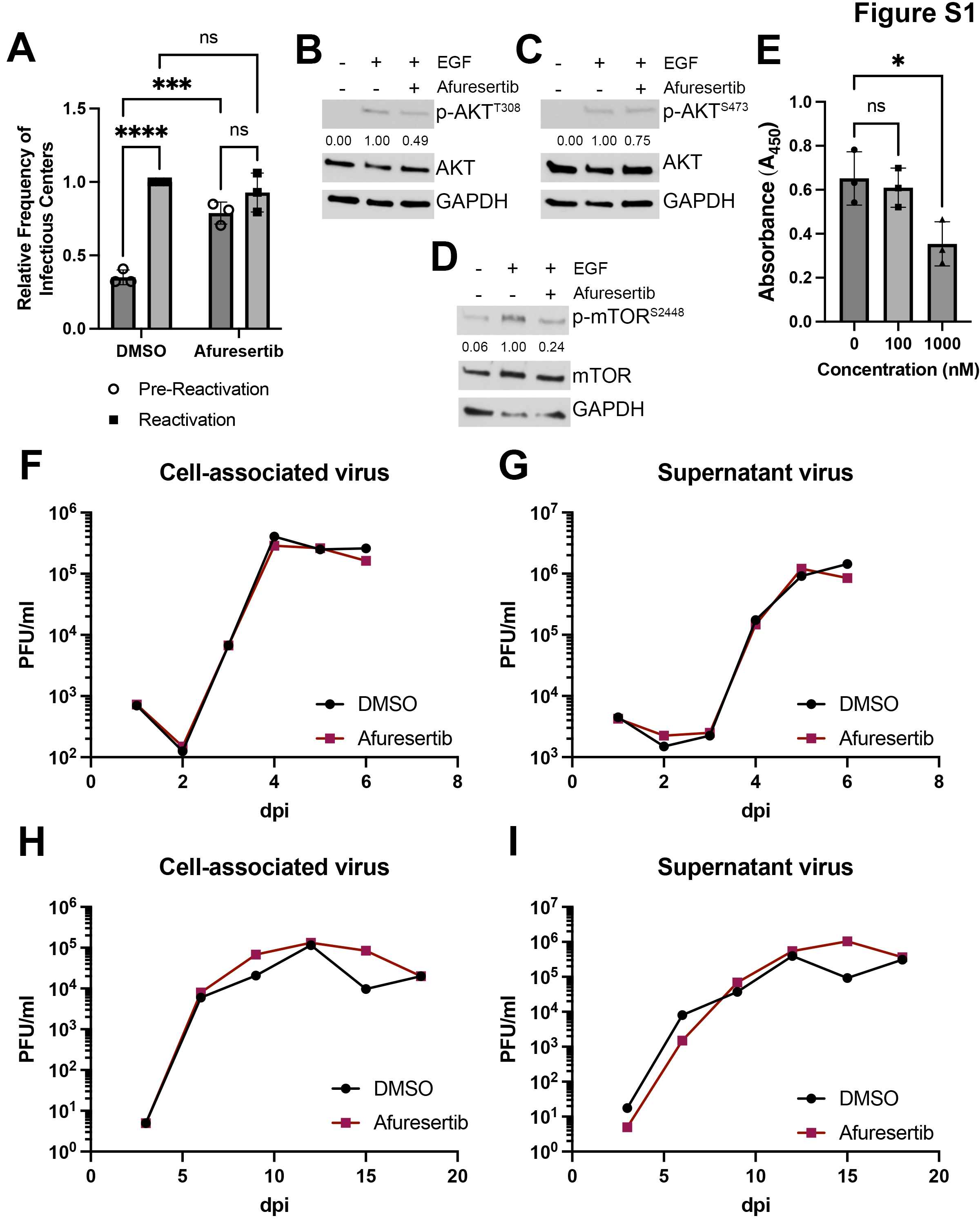

Supplement: S1 Fig — (A) hESC-derived CD34+ HPCs were infected with HCMV TB40/E-GFP at an MOI of 2 for 48hr and then sorted by FACS for viable, CD34+, GFP+ cells. Infected HPCs were maintained in LTBMC culture medium in transwells over stromal cells for 12 days to establish latency in the presence of Afuresertib (100 nM) or DMSO (control). Following the latency culture, cells were co-cultured in cytokine-rich media in an extreme limiting dilution assay (ELDA) to measure virus reactivation. An equal number of cells were mechanically disrupted and seeded in parallel to measure infectious virus present in the latency culture (pre-reactivation). At 21 days post-plating, the number of GFP+ wells were counted and the frequency of infectious center production was determined by ELDA software. Reactivation is shown as the relative frequency of infectious centers compared to DMSO control-treated cells from three independent experiments (***p<0.0005, ****p<0.0001 [two-way ANOVA with Tukey’s multiple comparison test]). (B-D) NHDFs were serum starved overnight and treated +/- 100nM Afuresertib. The next day, cells were stimulated +/- EGF for 15 minutes. Protein lysates were harvested and immunoblotted for p-Akt T308 (B), p-Akt S473 (C), or p-mTOR S2448 (D) as well as total Akt (B, C), total mTOR (D), and GAPDH. Quantification from one representative blot shows relative expression levels of p-AKT or p-mTOR compared to cells stimulated with EGF (normalized to GAPDH). (E) hESC-derived HPCs were incubated with the indicated concentrations of Afuresertib for 7 days. Cell viability was measured by WST-1 colorimetric assay (Roche) according to manufacturer’s instructions. Quantification shows absorbance at 450nm after background subtracting the value of media alone. Error bars represent standard deviation from triplicate samples from one representative experiment (*p<0.05 [one-way ANOVA with Tukey’s multiple comparison test]). (F-I) NHDFs were infected with WT TB40/E-GFP at an MOI of 3 for single-step (F, [file ppat.1012285.s001.tif]

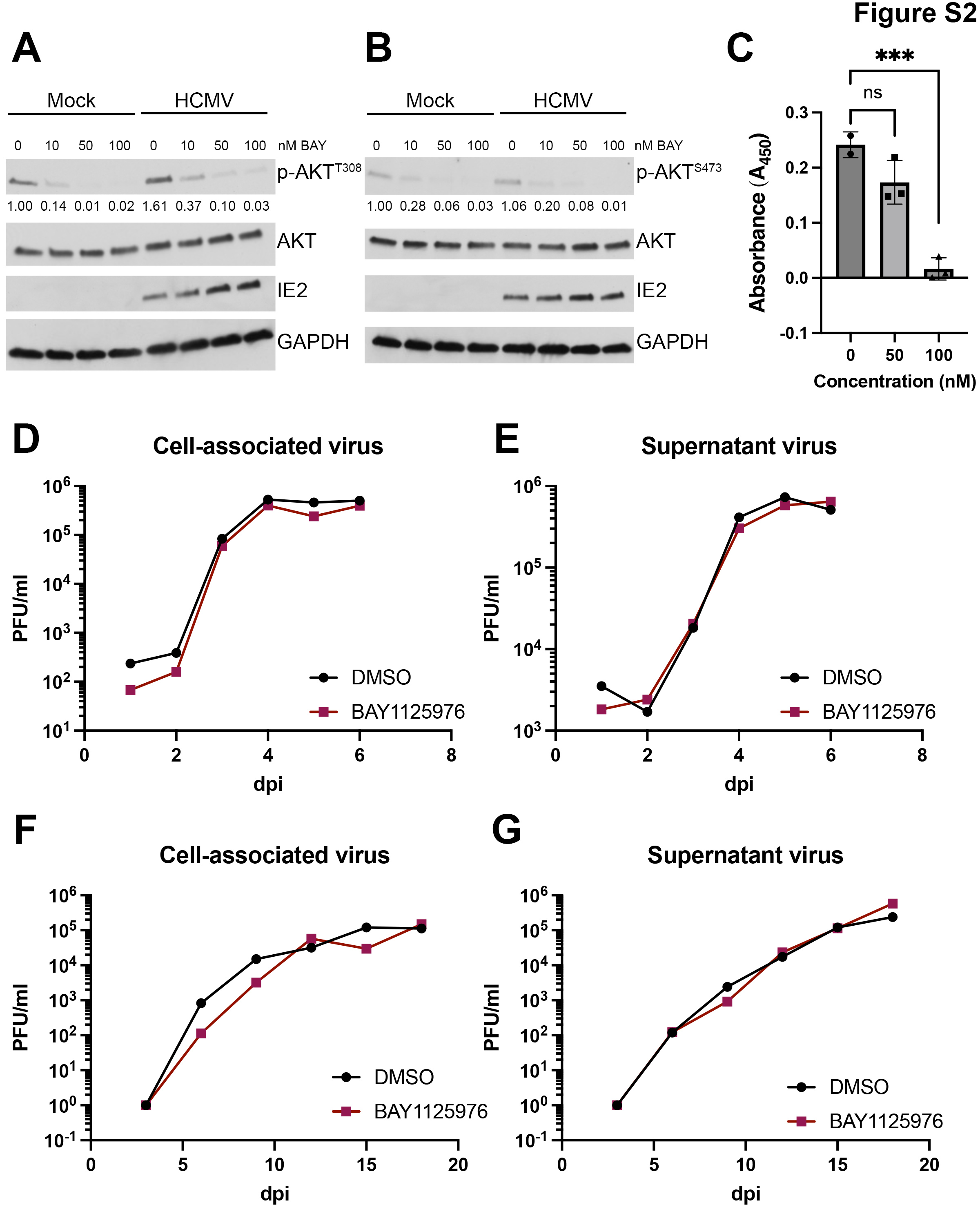

Supplement: S2 Fig — (A, B) NHDFs were infected with WT TB40/E-GFP (or Mock infected) at an MOI of 3 for 8hr and then were serum starved overnight in the presence of increasing concentrations of BAY1125976. At 24 hpi, cells were stimulated with EGF for 15 minutes. Lysates were then harvested and immunoblotted for p-Akt T308 (A) or p-Akt S473 (B) as well as total Akt, IE2, and GAPDH. Quantification from one representative blot shows relative expression levels of p-AKT compared to Mock-infected cells stimulated with EGF (normalized to GAPDH). (C) hESC-derived CD34+ HPCs were incubated with the indicated concentrations of BAY1125976 for 7 days. Cell viability was measured by WST-1 colorimetric assay (Roche) according to manufacturer’s instructions. Quantification shows absorbance at 450nm after background subtracting the value of media alone. Error bars represent standard deviation from triplicate samples from one representative experiment (*p<0.05 [one-way ANOVA with Tukey’s multiple comparison test]). (D-G) NHDFs were infected with TB40/E-GFP at an MOI of 3 for single-step (D, E) or an MOI of 0.01 for multistep (F, G) growth curves and treated +/- 50nM BAY1125976. PFU/ml values were quantified in duplicate from samples collected at the indicated time points for cell-associated (D, F) or supernatant (E, G) virus. (TIF) [file ppat.1012285.s002.tif]

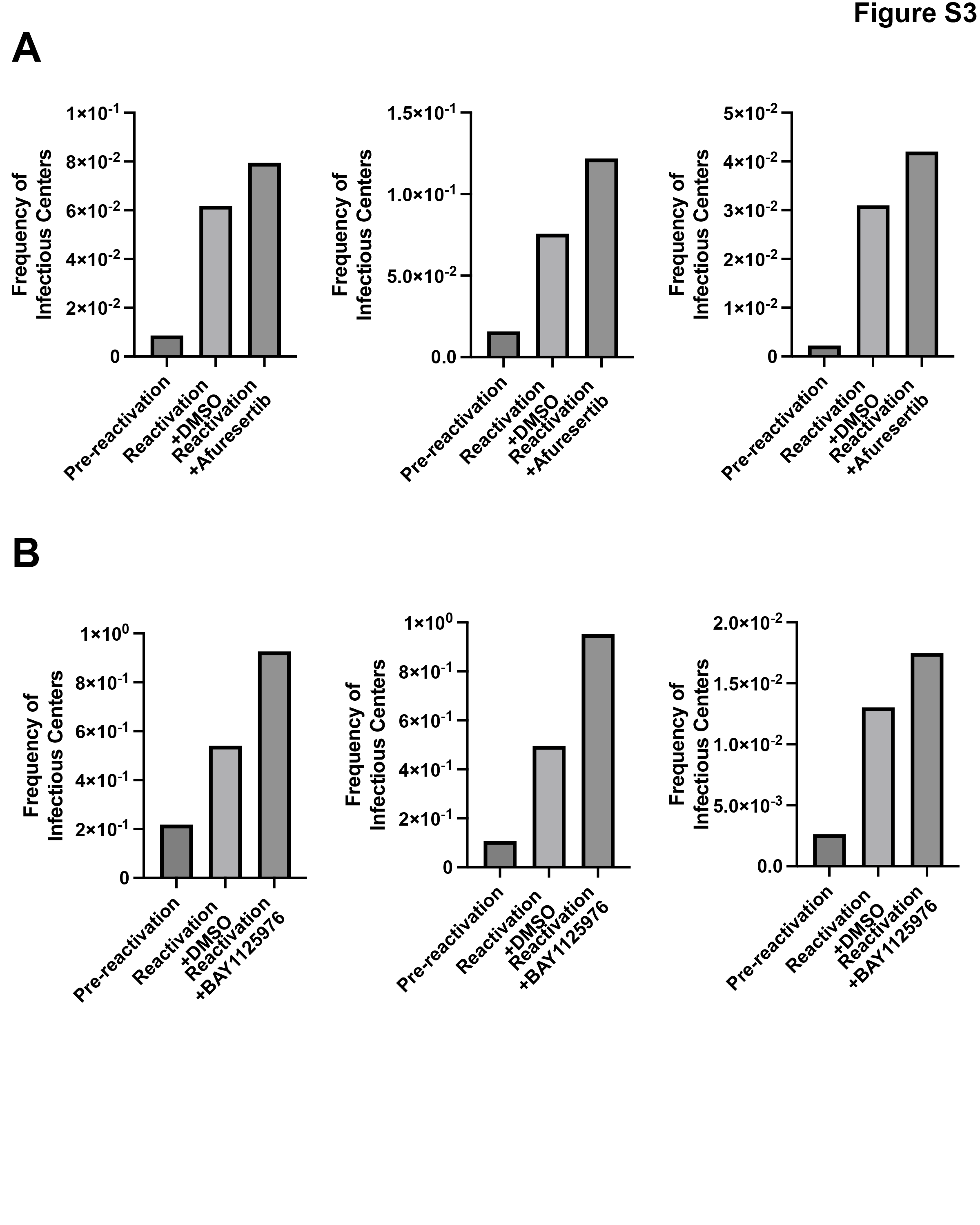

Supplement: S3 Fig — Individual experiments from Fig 1 show reactivation as the frequency of infectious centers for three replicate experiments for cells treated with Afuresertib (A) or BAY1125976 (B) during reactivation. (TIF) [file ppat.1012285.s003.tif]

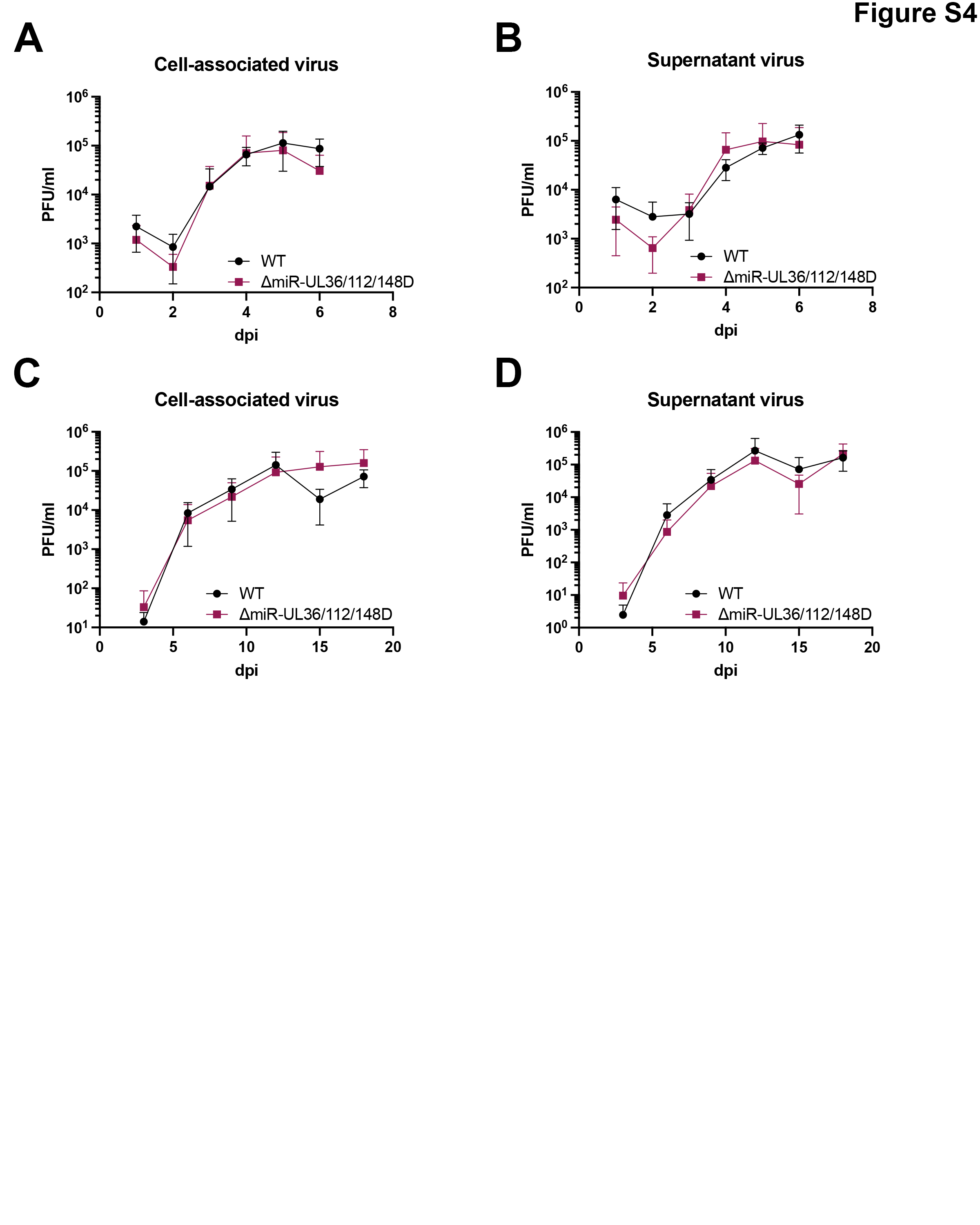

Supplement: S4 Fig — NHDFs were infected with WT TB40/E-GFP or ΔmiR-UL36/112/148D at an MOI of 3 for single-step (A, B) or an MOI of 0.01 for multistep (C, D) growth curves. PFU/ml values were quantified in duplicate from samples collected at the indicated time points for cell-associated (A, C) or supernatant (B, D) virus. (TIF) [file ppat.1012285.s004.tif]

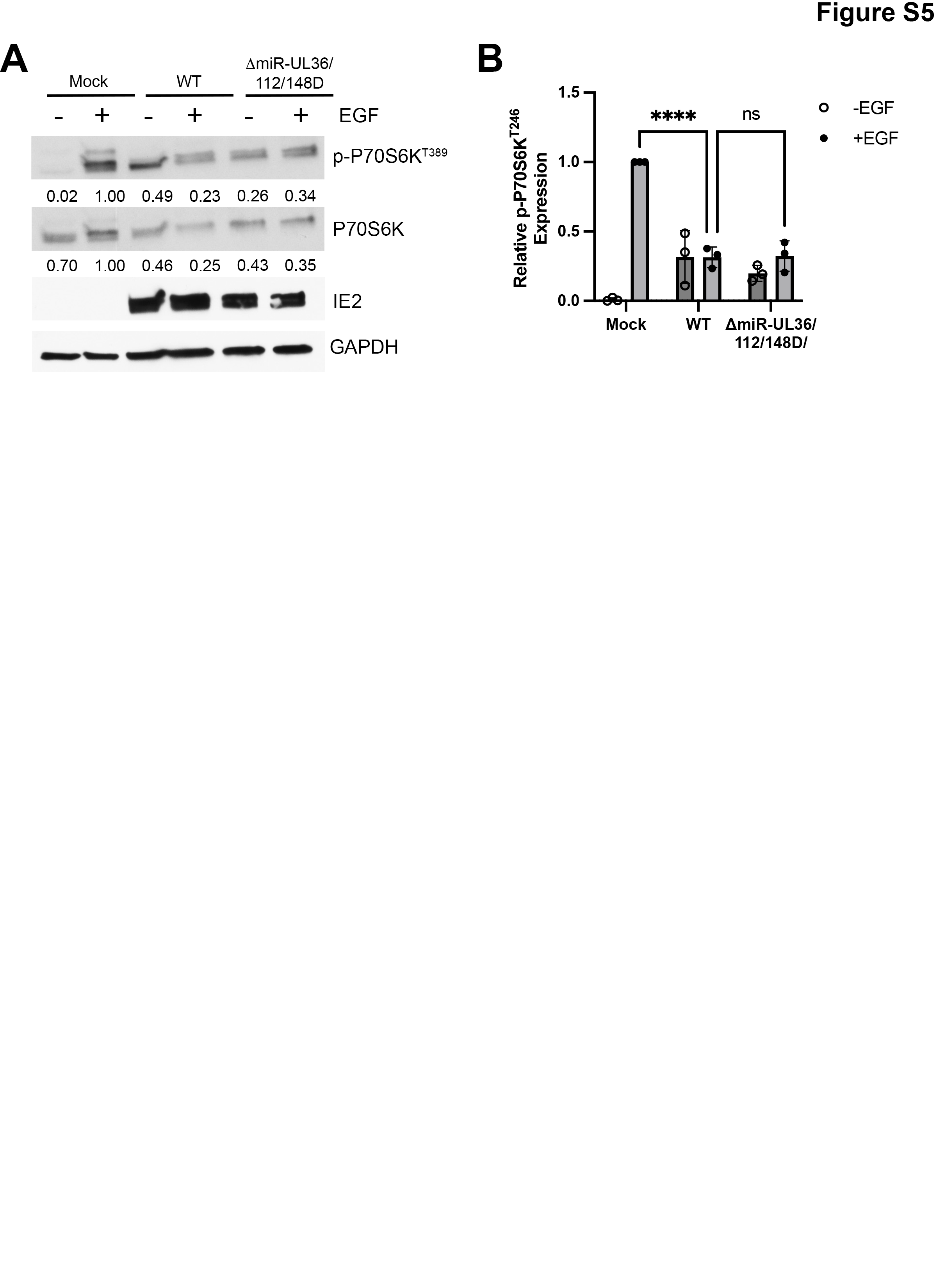

Supplement: S5 Fig — (A) NHDF were infected at an MOI of 3 with WT, ΔmiR-UL36/112/148D, or Mock infected for 48hr, serum starved overnight, and then stimulated +/-EGF for 15 minutes. Lysates were then harvested and immunoblotted for phosphorylated and total P70S6K as well as HCMV IE2 and GAPDH. Quantification from one representative blot shows relative expression levels of p-P70S6K and total P70S6K compared to Mock (normalized to GAPDH). (C, D) Quantification of (A, B), respectively, from three separate experiments (comparing +EGF conditions, ****p<0.0001 [two-way ANOVA with Tukey’s multiple comparison test]). (TIF) [file ppat.1012285.s005.tif]

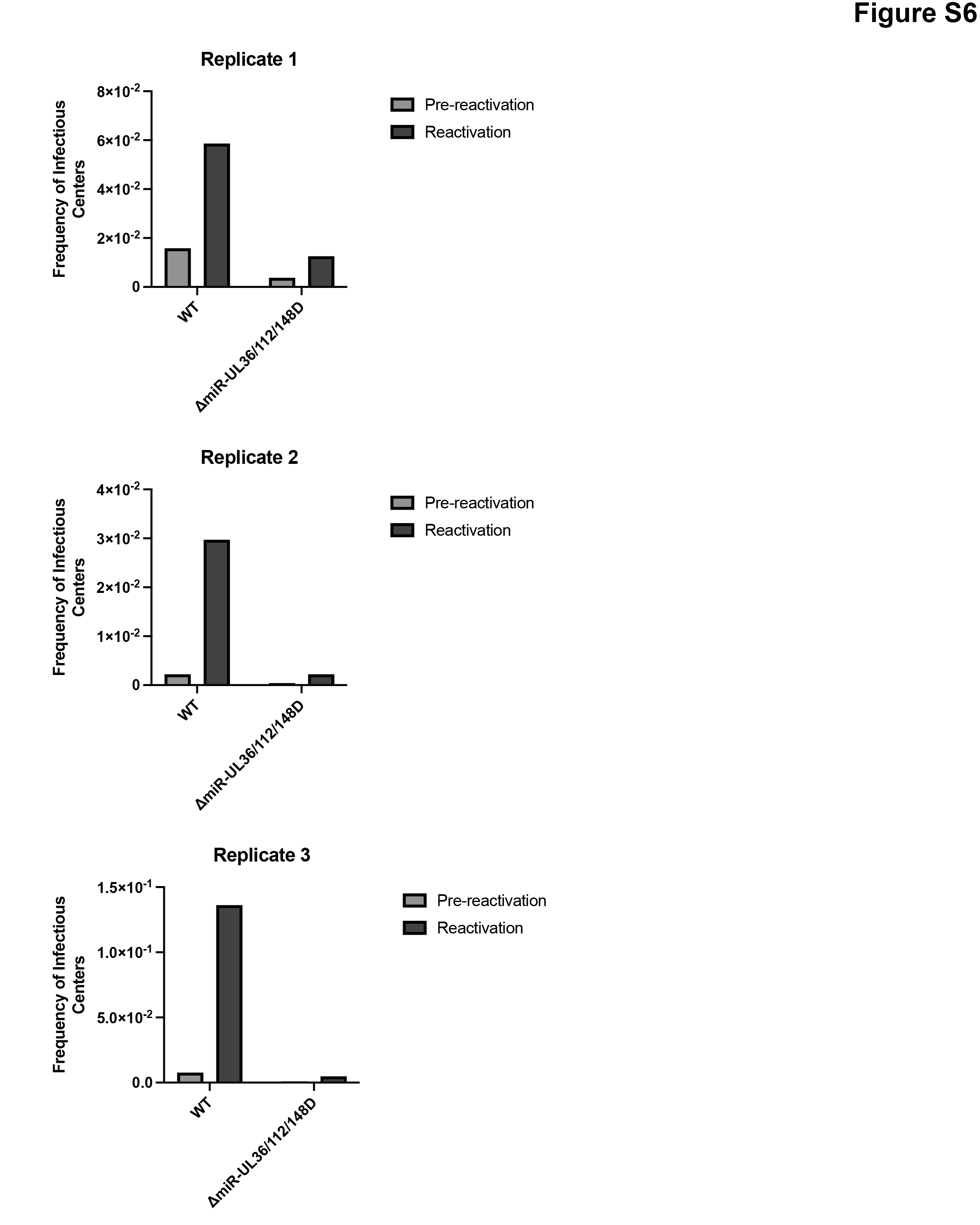

Supplement: S6 Fig — Individual experiments from Fig 7C show reactivation as the frequency of infectious centers for three replicate experiments. (TIF) [file ppat.1012285.s006.tif]

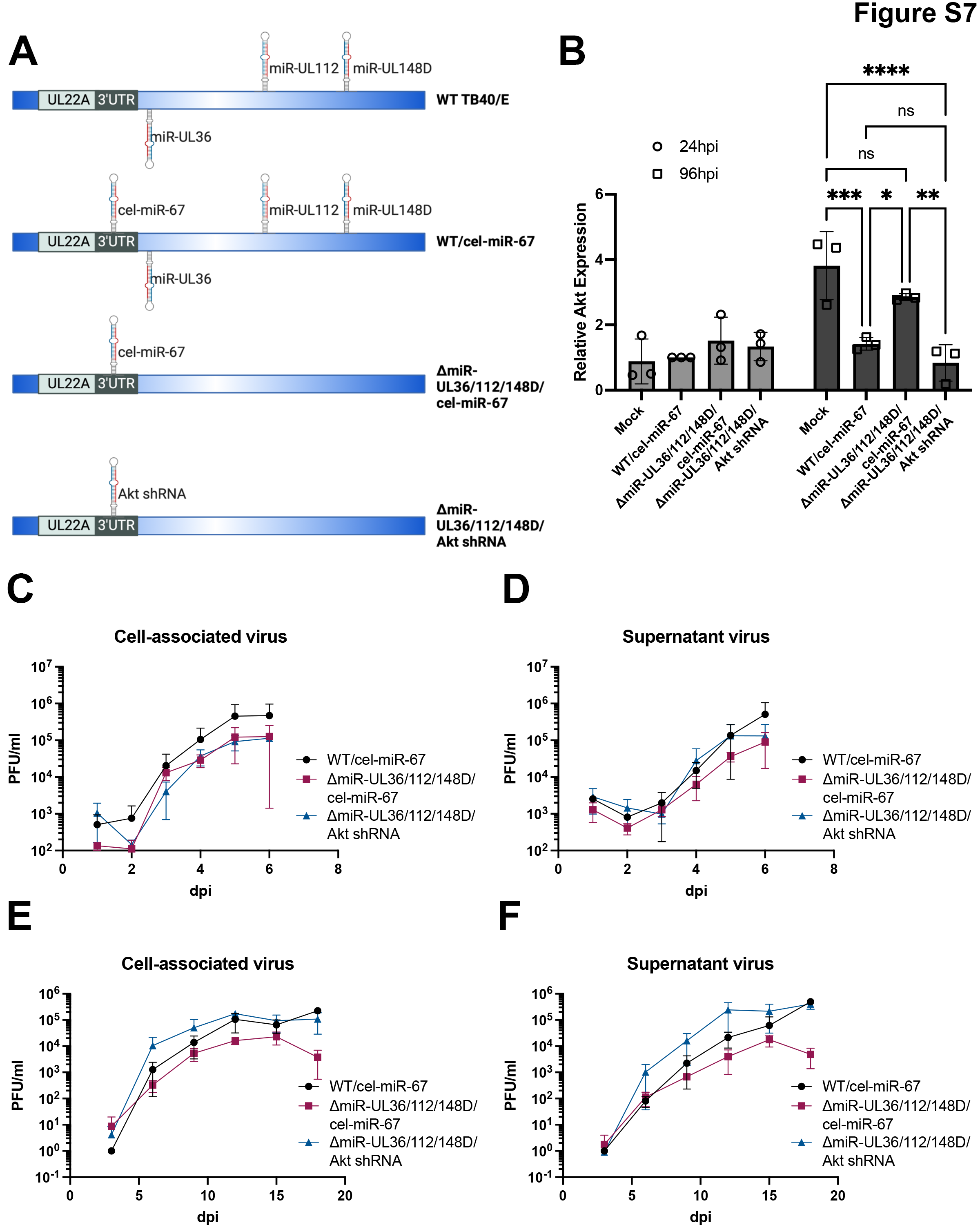

Supplement: S7 Fig — (A) Schematic of cel-miR-67 and Akt shRNA-expressing viruses. From top to bottom: WT TB40/E-GFP, WT TB40/E-GFP expressing C. elegans miR-67 (cel-miR-67) from the 3’UTR of UL22A (WT/cel-miR-67), ΔmiR-UL36/112/148D expressing cel-miR-67 (ΔmiR-UL36/112/148D/cel-miR-67), or ΔmiR-UL36/112/148D expressing an Akt shRNA from this same region (ΔmiR-UL36/112/148D/Akt shRNA). (B) NHDFs were infected at an MOI of 3 with WT/cel-miR-67, ΔmiR-UL36/112/148D/cel-miR-67, ΔmiR-UL36/112/148D/Akt shRNA, or Mock infected for 24 or 96hr after which RNA was harvested. Quantitative RT-PCR was performed using specific primers for Akt. Expression levels were normalized to GAPDH and compared to Mock (*p<0.05, **p<0.005, ***p<0.0005, ****p<0.0001 [two-way ANOVA with Tukey’s multiple comparison test]). (C-F) NHDFs were infected with WT/cel-miR-67, ΔmiR-UL36/112/148D/cel-miR-67, or ΔmiR-UL36/112/148D/Akt shRNA at an MOI of 3 for single-step (C, D) or an MOI of 0.01 for multistep (E, F) growth curves. PFU/ml values were quantified in duplicate from samples collected at the indicated time points for cell-associated (C, E) or supernatant (D, F) virus. (TIF) [file ppat.1012285.s007.tif]

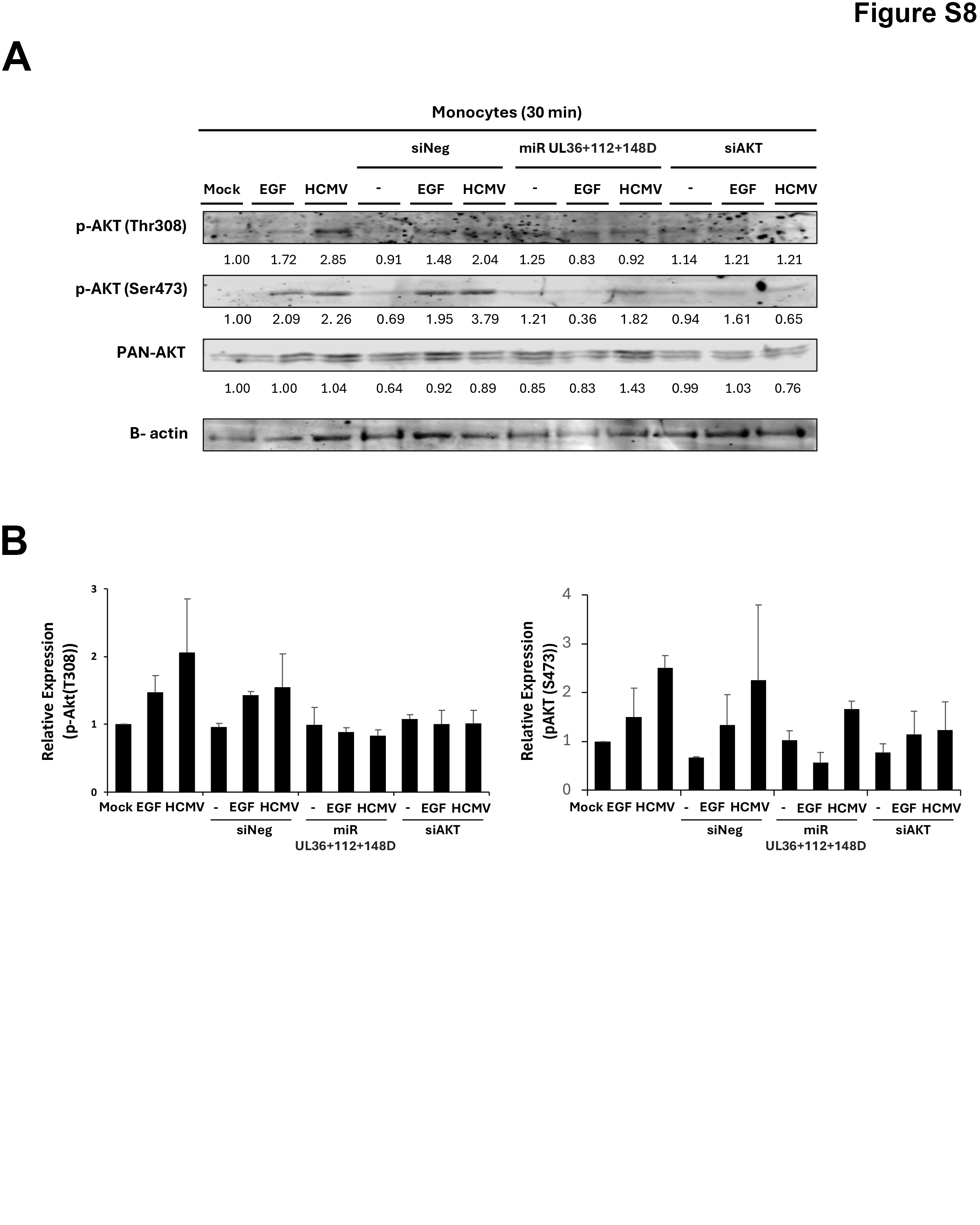

Supplement: S8 Fig — (A) Human monocytes were transfected with a negative control small interfering RNA (siRNA) (scrambled control; siNeg), a combination of miRNAs (miR-UL36+UL112+UL148D), or an siRNA targeting AKT (siAKT), and incubated for 48 hrs. 48 hrs after transfection, monocytes were infected with TB40/E at an MOI of 5 for 30 minutes or treated with hEGF for 30 minutes, and then protein lysates were harvested and immunoblotted for p-AKT (Ser473), p-AKT (Thr308), pan-AKT (PAN-AKT) and β-actin. (B) Relative band intensity was determined for pAKT (T308 and S473) compared to β-actin. A representative blot and band intensities are shown. 3 identical experiments with different human donors were performed and similar results were observed with all the donors. (TIF) [file ppat.1012285.s008.tif]

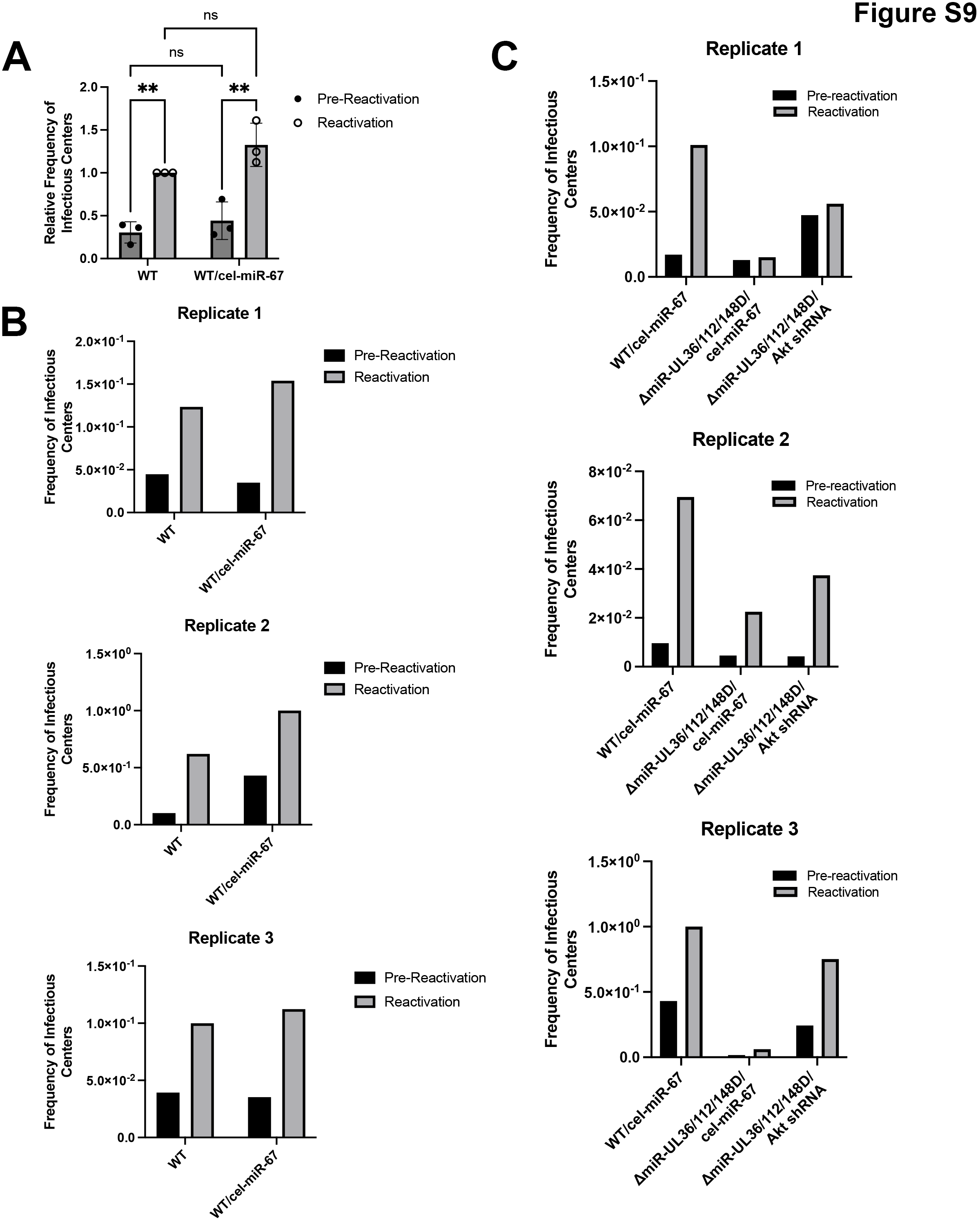

Supplement: S9 Fig — (A) hESC-derived CD34+ HPCs were infected with HCMV TB40/E-GFP or WT/cel-miR-67 at an MOI of 2 for 48hr and then sorted by FACS for viable, CD34+, GFP+ cells. Infected HPCs were maintained in LTBMC culture medium in transwells over stromal cells for 12 days to establish latency. Following the latency culture, cells were co-cultured in cytokine-rich media in an extreme limiting dilution assay (ELDA) to measure virus reactivation. An equal number of cells were mechanically disrupted and seeded in parallel to measure infectious virus present in the latency culture (pre-reactivation). At 21 days post-plating, the number of GFP+ wells were counted and the frequency of infectious center production was determined by ELDA software. Reactivation is shown as the relative frequency of infectious centers compared to DMSO control-treated cells from three independent experiments (**p<0.005 [two-way ANOVA with Tukey’s multiple comparison test]). (B) Individual experiments from S8A Fig show reactivation as the frequency of infectious centers for three replicate experiments. (C) Individual experiments from Fig 8D show reactivation as the frequency of infectious centers for three replicate experiments. (TIF) [file ppat.1012285.s009.tif]

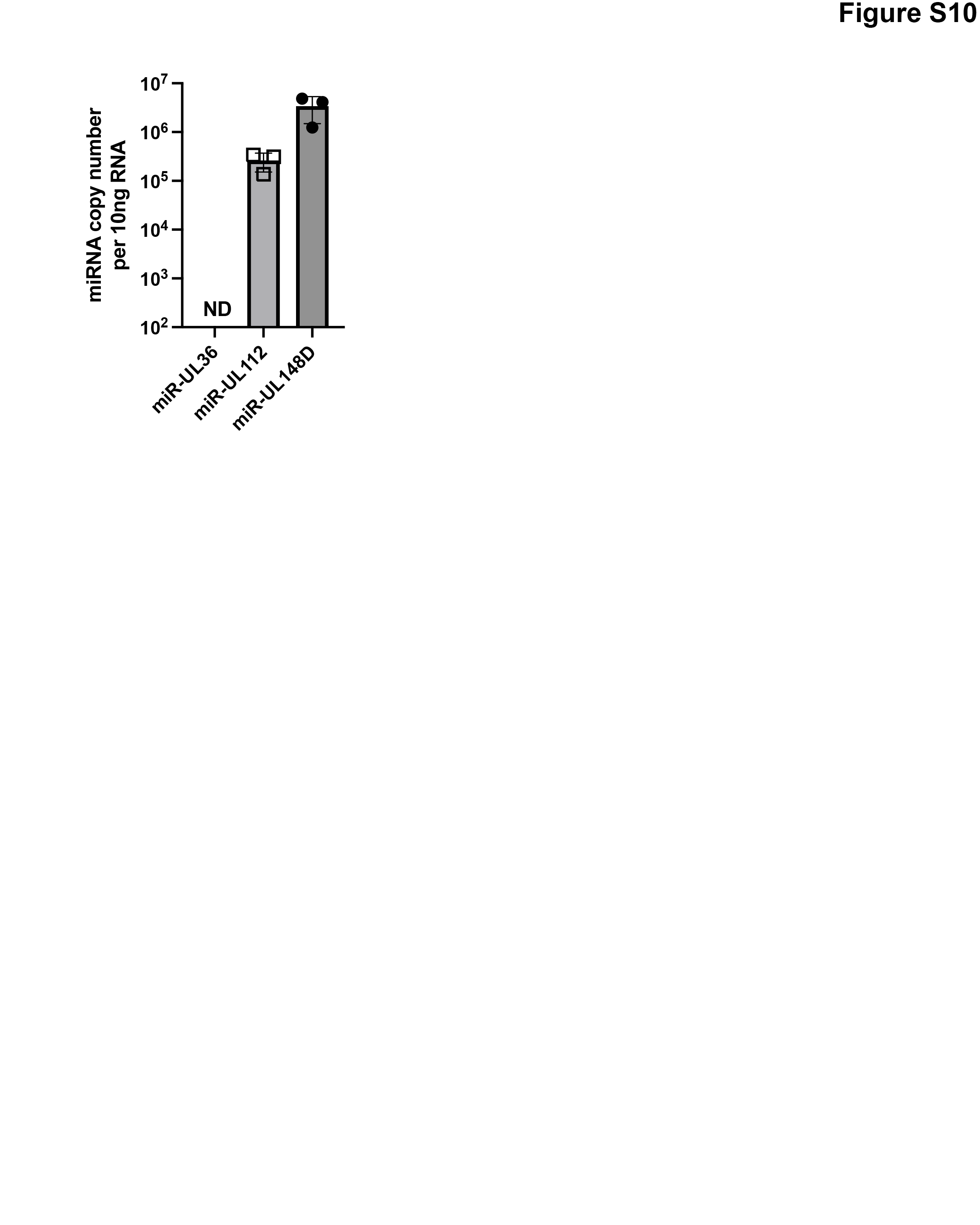

Supplement: S10 Fig — hESC-derived CD34+ HPCs were infected at an MOI of 2 for 48 hours, then FACS-isolated for viable, CD34+, GFP+ HPCs. Sorted cells were cultured under latency conditions for 12 days to establish latency and HCMV miRNA levels were detected in 10ng RNA from infected cells by stem-loop qRT-PCR. (TIF) [file ppat.1012285.s010.tif]

**Figure 2**

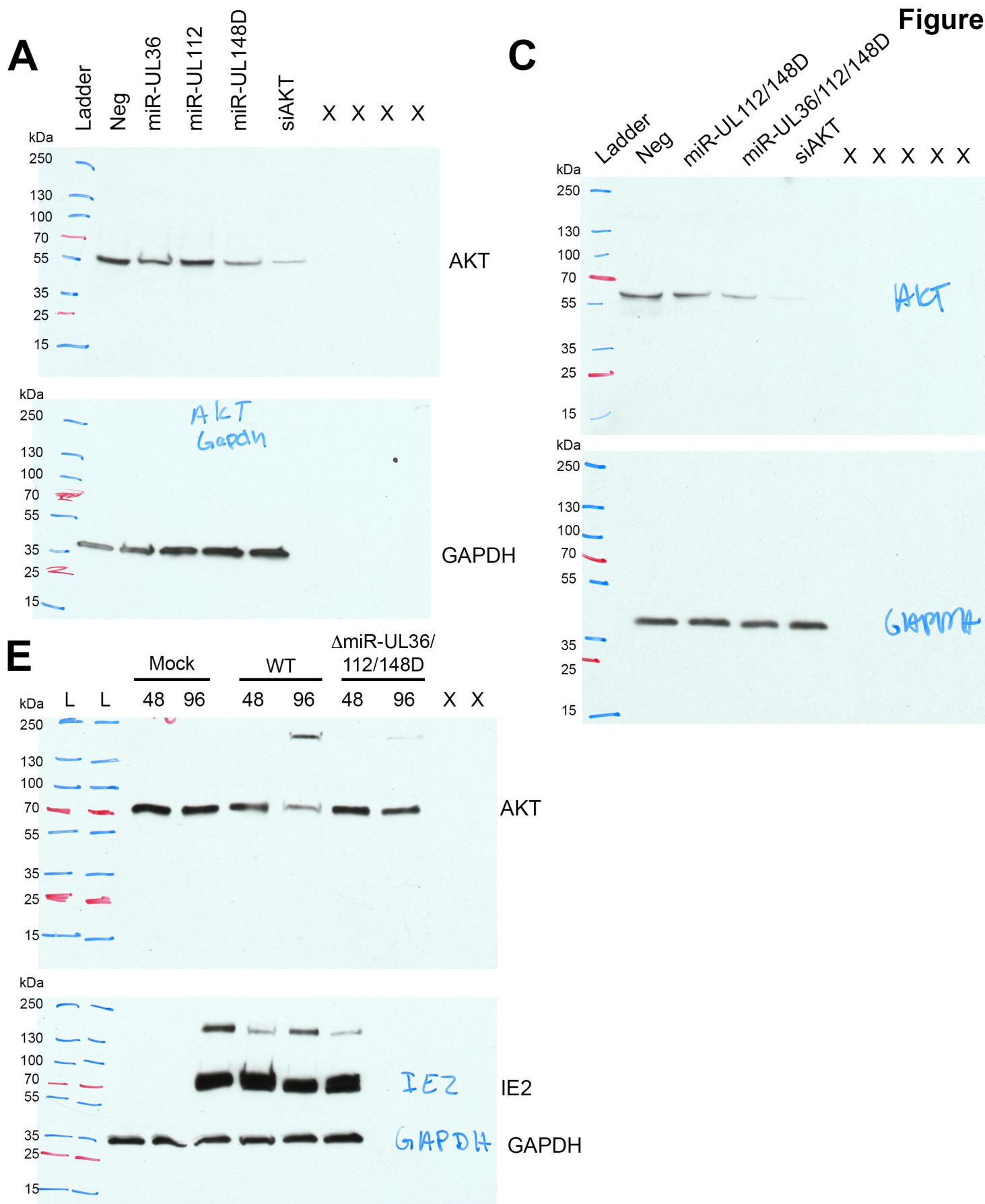

**F**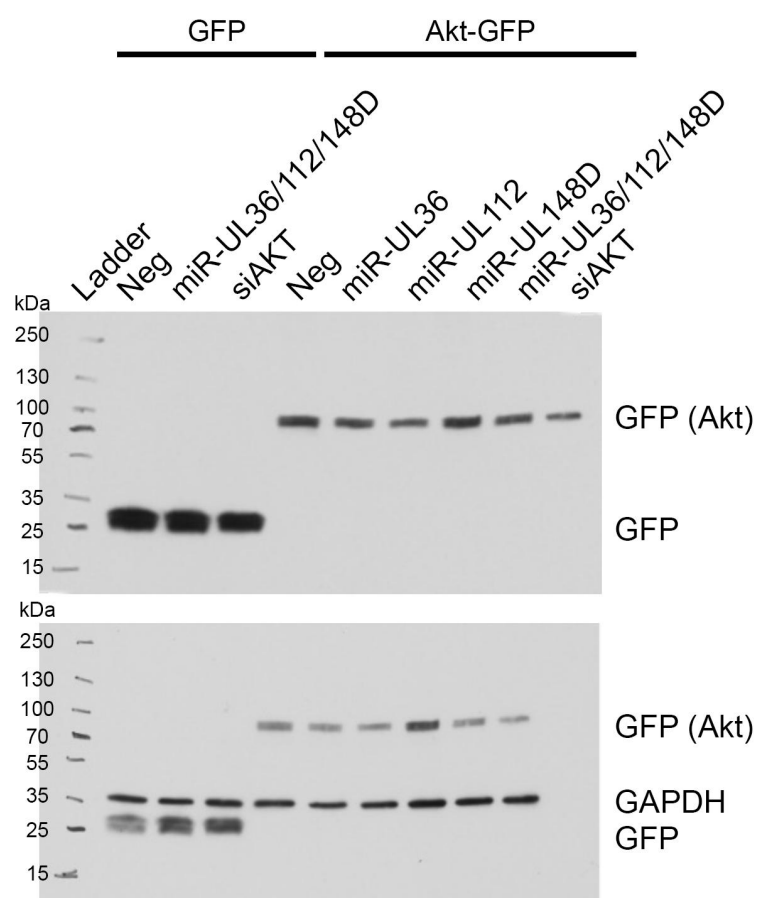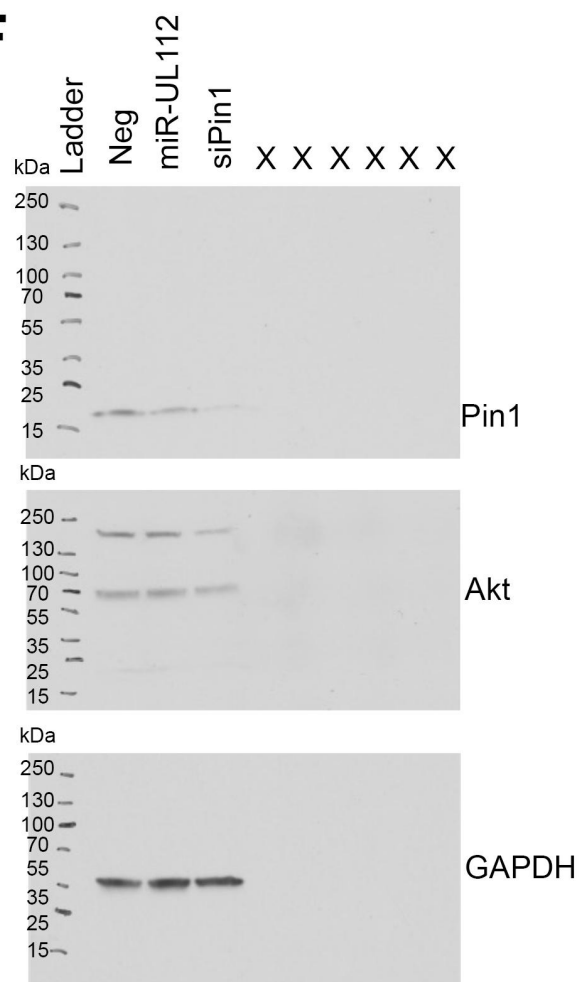

4A

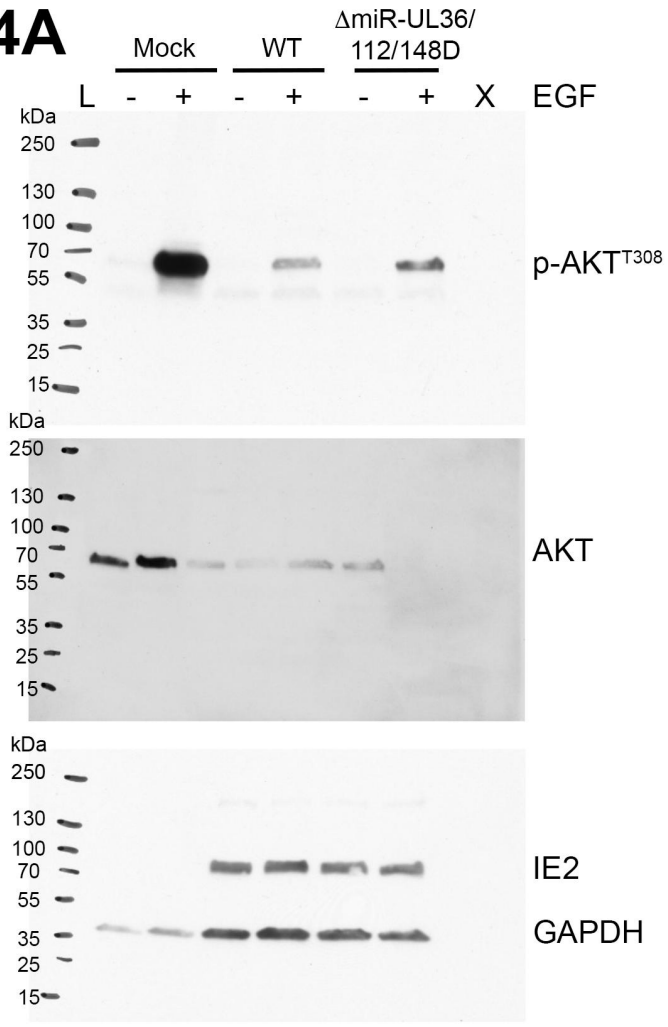

4B/5E

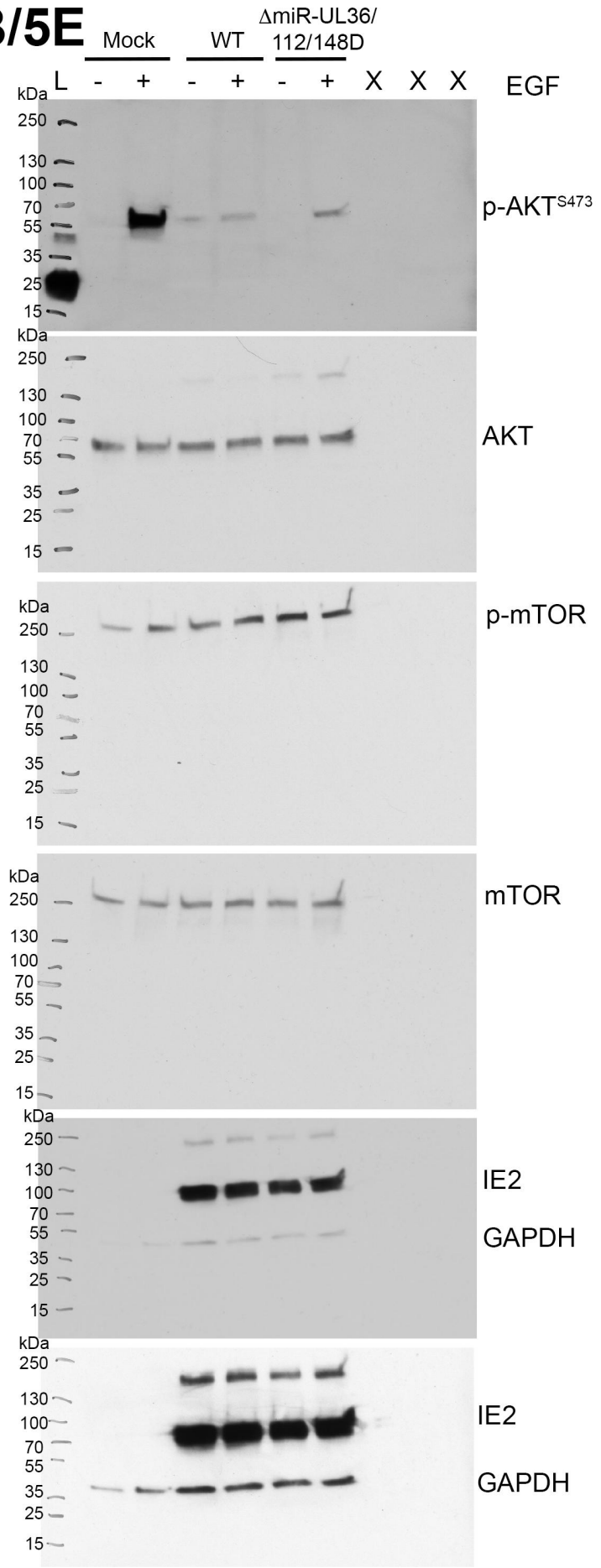

**Figure 5**

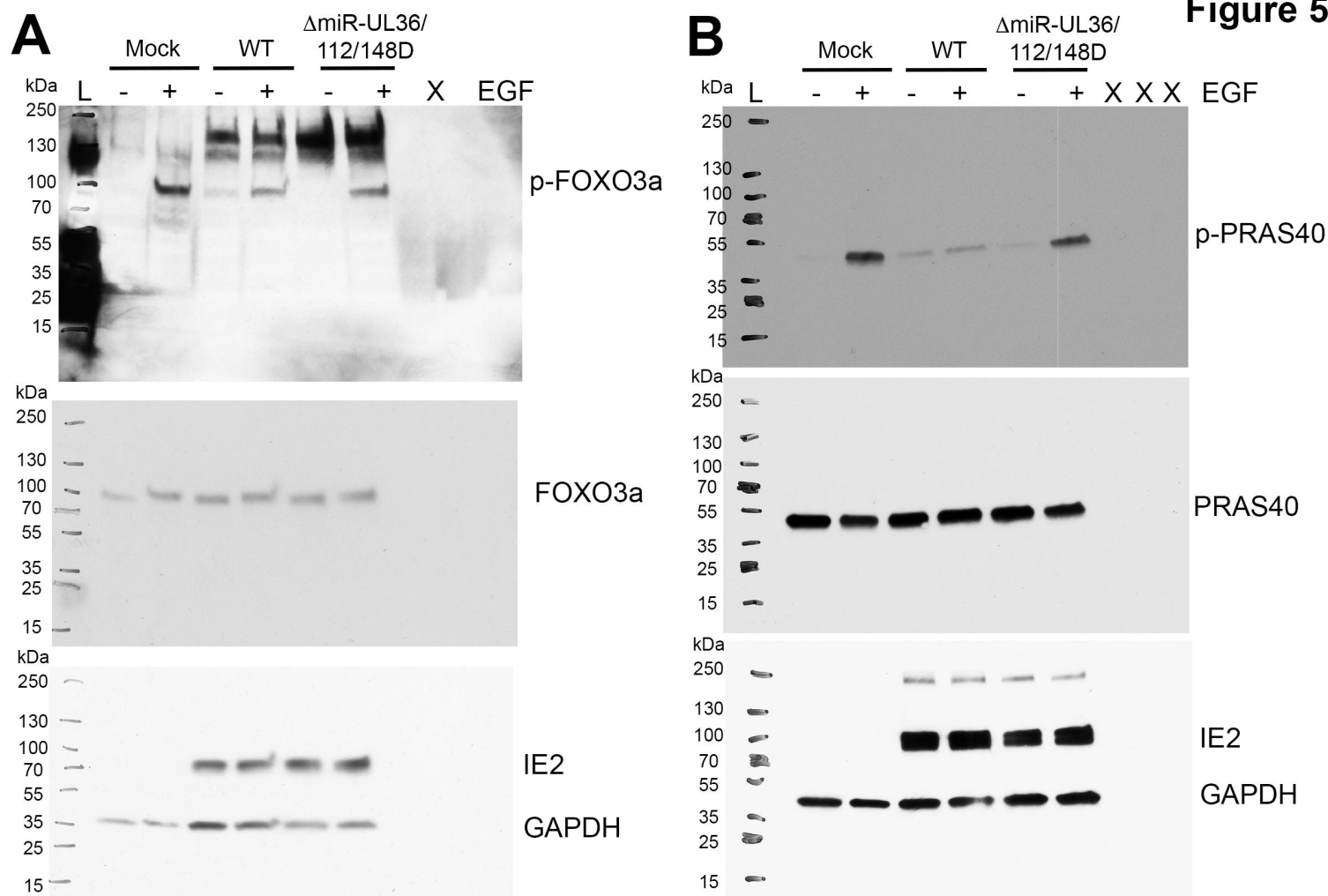



**Figure 8**

**A**

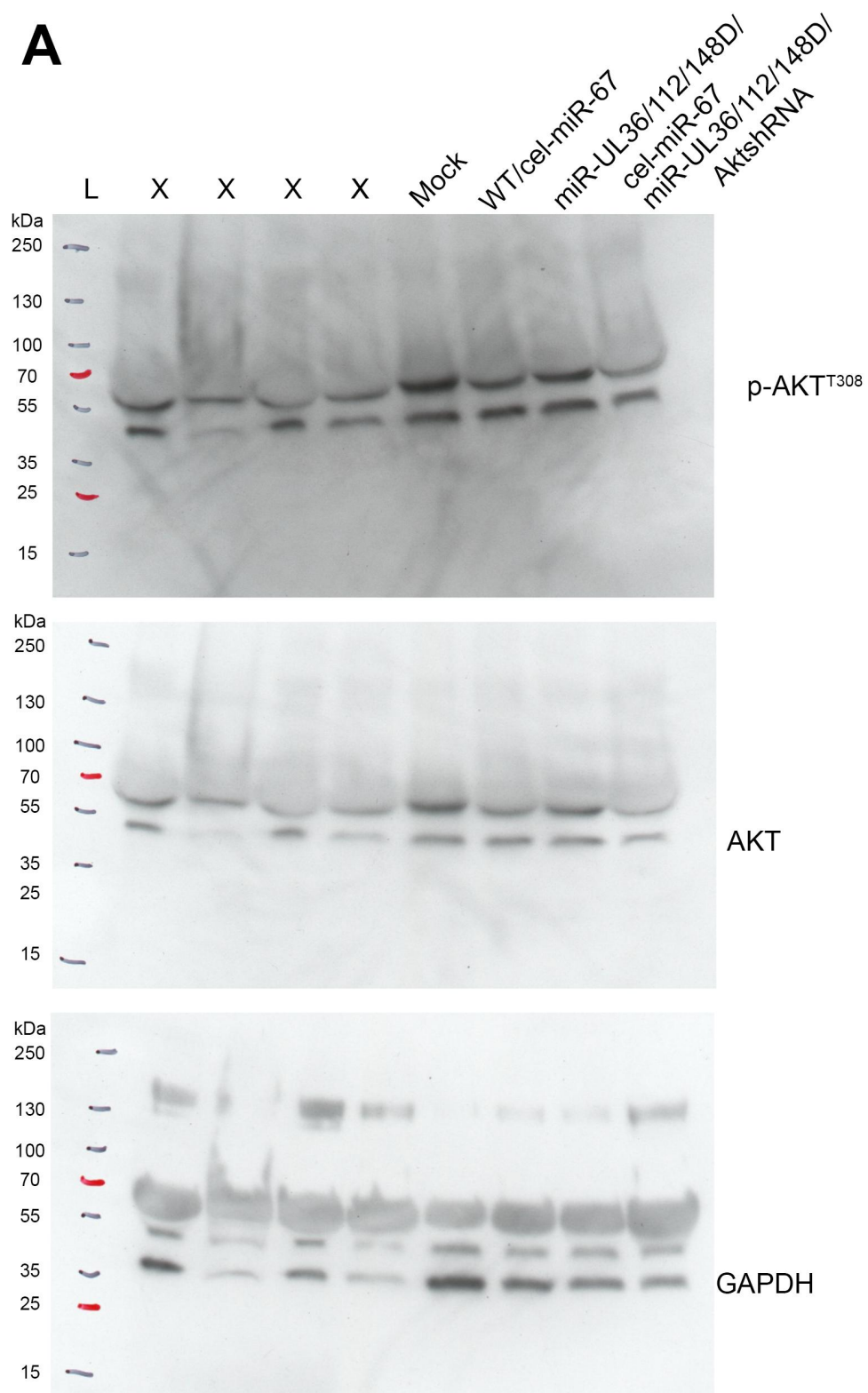



**Figure S2**

**A**

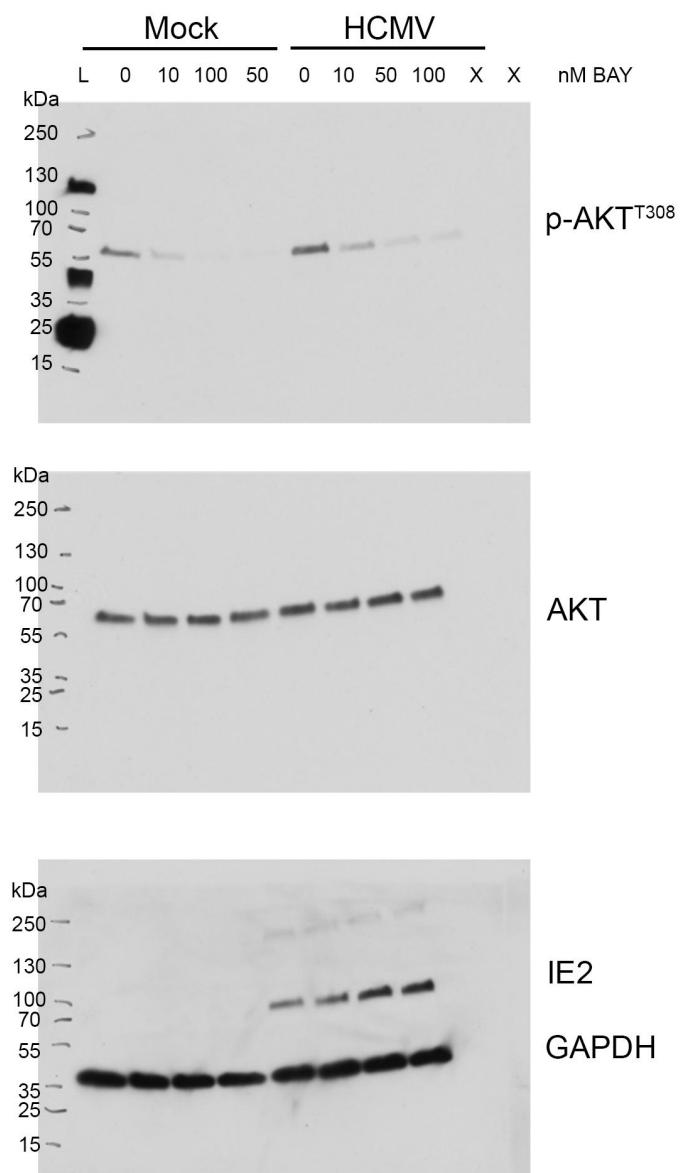

**B**

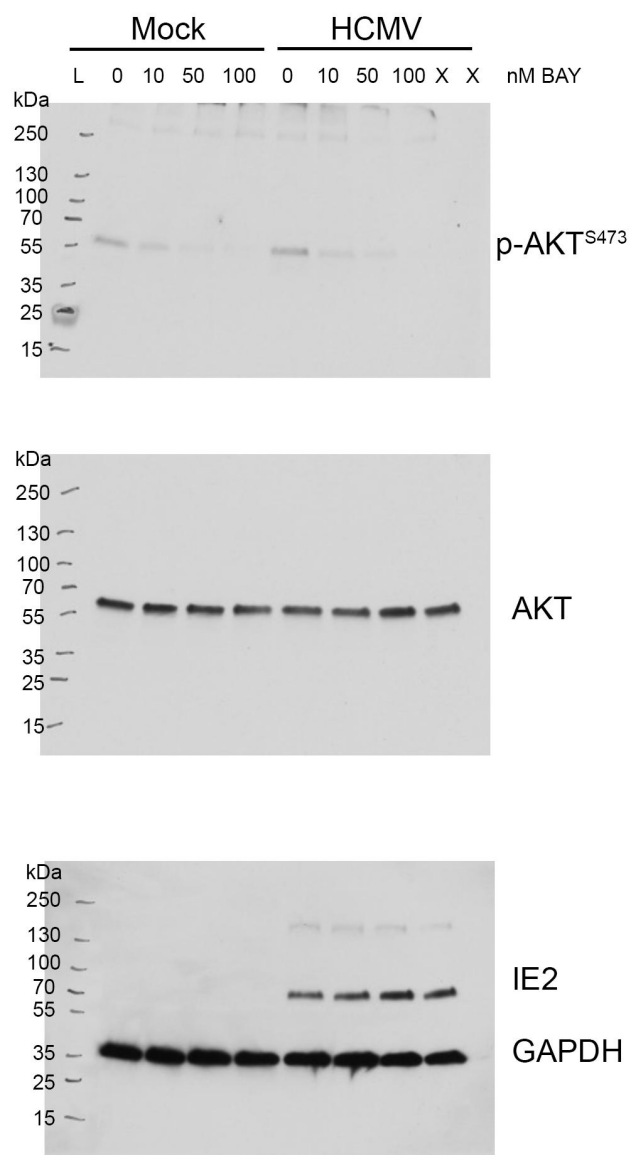

Supplement: S1 Data — Raw immunoblot data associated with main and supplemental figures. (PDF) [file ppat.1012285.s011.pdf]
